# Pediatric glioma histone H3.3 K27M/G34R mutations drive abnormalities in PML nuclear bodies

**DOI:** 10.1186/s13059-023-03122-5

**Published:** 2023-12-08

**Authors:** Hsiao P. J. Voon, Linda Hii, Andrew Garvie, Maheshi Udugama, Brian Krug, Caterina Russo, Anderly C. Chüeh, Roger J. Daly, Alison Morey, Toby D. M. Bell, Stephen J. Turner, Joseph Rosenbluh, Paul Daniel, Ron Firestein, Jeffrey R. Mann, Philippe Collas, Nada Jabado, Lee H. Wong

**Affiliations:** 1 Cancer Program, Department of Biochemistry and Molecular Biology, Biomedicine Discovery Institute, Monash University, Clayton, Australia; 2 Department of Human Genetics, McGill University, Montreal, QC Canada; 3 Department of Microbiology, Monash University, Clayton, VIC Australia; 4 School of Chemistry, Monash University, Clayton, VIC Australia; 5 Hudson Institute of Medical Research, Clayton, VIC Australia; 6 Department of Molecular and Translational Science, Monash University, Clayton, VIC Australia; 7 Department of Anatomy and Developmental Biology, Monash University, Clayton, VIC Australia; 8 Department of Molecular Medicine, Institute of Basic Medical Sciences, Faculty of Medicine, University of Oslo, 0317 Oslo, Norway; 9 Department of Immunology and Transfusion Medicine, Oslo University Hospital, 0424 Oslo, Norway; 10 Division of Experimental Medicine, McGill University, Montreal, QC Canada; 11 Department of Paediatrics, Research Institute of the McGill University Health Centre, Montreal, QC Canada

**Keywords:** Histone variant H3.3, Pediatric glioma, PML bodies, Arsenic trioxide

## Abstract

**Background:**

Point mutations in histone variant H3.3 (H3.3K27M, H3.3G34R) and the H3.3-specific ATRX/DAXX chaperone complex are frequent events in pediatric gliomas. These H3.3 point mutations affect many chromatin modifications but the exact oncogenic mechanisms are currently unclear. Histone H3.3 is known to localize to nuclear compartments known as promyelocytic leukemia (PML) nuclear bodies, which are frequently mutated and confirmed as oncogenic drivers in acute promyelocytic leukemia.

**Results:**

We find that the pediatric glioma-associated H3.3 point mutations disrupt the formation of PML nuclear bodies and this prevents differentiation down glial lineages. Similar to leukemias driven by PML mutations, H3.3-mutated glioma cells are sensitive to drugs that target PML bodies. We also find that point mutations in IDH1/2—which are common events in adult gliomas and myeloid leukemias—also disrupt the formation of PML bodies.

**Conclusions:**

We identify PML as a contributor to oncogenesis in a subset of gliomas and show that targeting PML bodies is effective in treating these H3.3-mutated pediatric gliomas.

**Supplementary Information:**

The online version contains supplementary material available at 10.1186/s13059-023-03122-5.

## Background

Point mutations in histone variant H3.3 are common drivers of pediatric gliomas [1, 2]. A glycine to arginine substitution at position 34 of H3.3 (H3.3 G34R) occurs in ~ 30% of pediatric glioblastomas [3]. A second lysine to methionine substitution at position 27 (K27M) is the defining feature of a group of tumors recently classified as “diffuse midline gliomas, H3K27M,” which includes tumors previously known as diffuse intrinsic pontine glioma (DIPG) [4].

Histones are the protein component of nucleosomes, which form the basic repeated structural unit of chromosomes. Each nucleosome consists of ~ 146 bp of DNA wrapped around a histone octamer comprised of two units each of histone H2A, H2B, H3, and H4. The overwhelming bulk of nucleosomes are comprised of “canonical” replication-dependent histones (e.g., H3.1/2) which are synthesized only during S-phase of the cell cycle when nucleosomes are rapidly assembled behind the DNA replication fork [5]. This coordinated high-level synthesis of histones is facilitated by multicopy histone H3-encoding genes—10 and 3 copies encoding H3.1 and H3.2 respectively [5]. In addition to the canonical histones, histone variants can also be incorporated into nucleosomes under certain circumstances. For example, histone H3.3 is a replication-independent H3 variant which is able to replace canonical H3.1/2 histones that are displaced outside of S-phase [6, 7].

Due to this pattern of incorporation, the H3.3 variant is largely linked to sites of active transcription such as the promoters of expressed genes, where it replaces canonical histones which have been disrupted by the passage of DNA polymerase. In addition, H3.3 is also involved in a range of genomic processes including maintenance of heterochromatin and integrity of repetitive DNA [8–13], cellular memory and differentiation, and development [14–17]. Overall, H3.3 accounts for a small fraction of the total pool of H3 [18], reflected in the low copy number of H3.3 genes (2 copies, *H3F3A H3F3B)*, compared to the 13 canonical H3 genes [5]. Despite the relative paucity of H3.3 and the disparity in copy number, H3.3 is far more frequently mutated in pediatric gliomas than the canonical H3.1/2 [1, 2, 19]. Indeed, all G34R mutations and 83% of the K27M mutations discovered thus far have been found on histone H3.3 [19], strongly suggesting that H3.3-specific pathways are involved in the development of pediatric gliomas.

H3.3 substitution point mutations are known to trigger diverse and widespread alterations in chromatin profiles. For example, H3.3 K27M has been reported to interfere with H3K27me3 [20–22], H3K27ac [23–25], H3K36me2 [26, 27], H3K4me3 [28], and DNA methylation [21]. Similarly, the H3.3 G34 mutations reportedly disrupt H3K36me3 [22, 29], H3K9me3 [29], H3K27me3 [30], and DNA methylation [31]. These observations suggest that the H3.3 point mutations have a very strong dominant-negative effect across a range of chromatin modifiers, but the mechanism which underlies this phenomenon is currently unclear. Furthermore, there are no clear correlations between sites of H3.3 enrichment and chromatin changes, suggesting that the mutant histone may exert effects on chromatin proteins independently of chromatinization.

One possible explanation for this phenomenon could lie in the chaperone complexes which distinguish H3.1/2 from H3.3. Histone H3.3 differs from H3.1/2 by only 4/5 amino acid residues including an Ala-Ala-Ile-Gly (AAIG) motif that mediates interaction with DAXX, an H3.3-specific chaperone [32]. DAXX forms a complex with the ATRX (α-thalassaemia with mental retardation, X-linked) chromatin remodeller [32], and this complex is also frequently mutated in gliomas [19], strongly suggesting that the ATRX/DAXX/H3.3 axis is pivotal in these cancers. Interestingly, this ATRX/DAXX/H3.3 complex is strongly associated with nuclear compartments known as promyelocytic nuclear bodies (PML-NBs) [33, 34], which is a frequently mutated oncogenic driver in acute promyelocytic leukemias [35]. PML-NBs are essential for regulating the function of the ATRX/DAXX complex, and the incorporation of H3.3 into heterochromatin domains such as the telomeres [33, 34, 36–38].

PML-NBs are naturally occurring membrane-less nuclear compartments which are involved in a range of processes including genome regulation, differentiation, and apoptosis [39]. These globular structures range from 0.1 to 1 µM in diameter and are comprised of a protein-dense inner core of nuclear proteins, encapsulated by an outer shell of oligomerized PML proteins [40, 41]. Both PML-NBs and associated proteins are highly dynamic [42] and therefore difficult to capture and purify [41]. Nonetheless, over 100 PML-associated proteins have been identified, including DAXX [34, 43], ATRX [34, 44], H3.3 [33, 36, 45], and other H3.3 chaperones [37, 46, 47]. In addition, a range of chromatin modifiers also localize to PML-NBs including histone lysine methyltransferases (e.g., SETDB1 [48], PRC2 [48]), acetyltransferases, deacetylases, and DNA demethylases (reviewed in [39]).

The *PML* gene was named for its role in a subtype of myeloid leukemias called acute *p*ro*m*yelocytic *l*eukemia (APL) [35]. Almost all (98%) APLs result from a fusion mutation between the *PML* and retinoic acid receptor α (*RARA*) genes [49], which causes distinct alterations in PML-NBs. In normal myeloid cells, PML has a punctate staining pattern with ~ 10–20 discrete foci of around 0.2–0.3 µm in size [50]. PML-RARα diffuses these PML-NBs into fine (< 0.1 µm) and abundant (> 100) speckles in APL [50]. Disrupting PML-NBs is thought to drive oncogenesis in APL by simultaneously blocking differentiation and preventing apoptosis, resulting in continued proliferation of stem-cell like progenitors [51].

Strikingly, H3.3-mutated gliomas exhibit a very similar phenotype of stalled differentiation [52], indicating H3.3 mutations may affect PML-NBs. Furthermore, we recently demonstrated that the H3.3 G34R mutation contributes to the formation of abnormal PML-NBs in pediatric gliomas with the *A*lternative *L*engthening of *T*elomeres (ALT) phenotype [53]. The telomeres of ALT cancers are encapsulated within abnormally large PML-NBs known as ALT-associated PML bodies (APBs) [54, 55] and we found that the combination of ATRX KO and H3.3 G34R mutations were necessary for APB formation [53]. Given the strong association between ATRX/H3.3 and PML-NBs, we sought to determine if pediatric glioma H3.3 mutations might exert oncogenic effects through disruption of PML-NBs, as is the case in APL.

We find that H3.3 point mutations interfere with the formation of PML-NBs and, much like APL, these PML defects contribute to blocked differentiation in pediatric gliomas. Consistent with this, histone-mutated gliomas are also highly sensitive to treatment with arsenic trioxide, which targets PML-NBs, showing that this pathway can be targeted for therapy. Interestingly, myeloid and glial malignancies also have frequent mutations in IDH1/2, and we have previously linked these mutations to ALT-positive cancers [53]. We find that IDH1 R132H point mutations also disrupt PML-NBs, suggesting that defective PML-NBs are a common oncogenic driver in gliomas and myeloid leukemias.

## Results

### Histone H3.3 is associated with PML across the genome

**Fig. 1:**
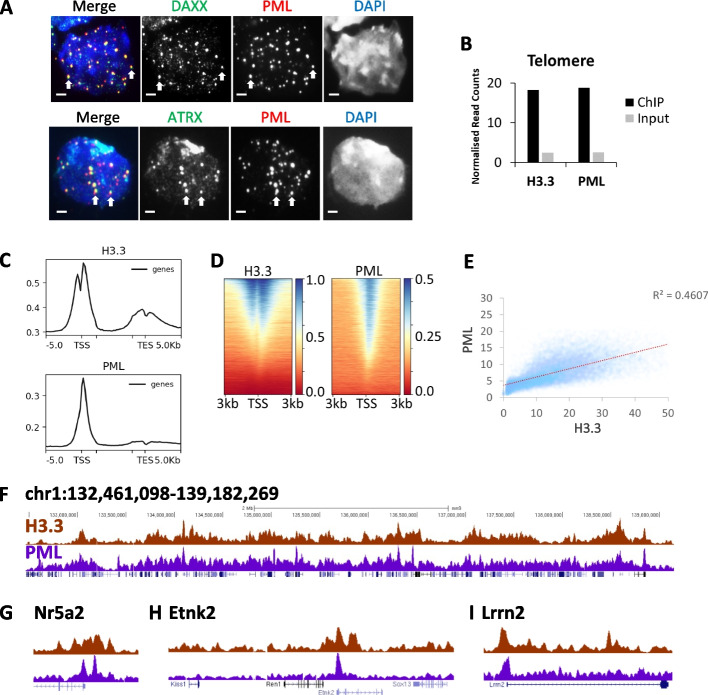
Histone H3.3 co-localizes with PML across the genome.** A** Immunofluorescence of PML (red) and DAXX (green) (top panel), and PML (red) and ATRX (green) (bottom panel) in WT mouse ES cells. Scale bar 2 µM.** B** H3.3 and PML ChIP-sequencing and input reads which map to telomere repeats. Bars represent ChIP-sequencing reads of H3.3 and PML in mouse ES cells normalized to total read count. Input sequencing shown as a control. **C** Composite profile of H3.3 and PML ChIP-seq reads across genes in mouse ES cells. **D** Heatmap of H3.3 and PML ChIP-seq reads at individual promoters [56], sorted by H3.3 enrichment. **E** Scatter plot of H3.3 and PML ChIP-seq reads which mapped to individual promoters, normalized for total read counts. **F** Representative UCSC genome browser profile of H3.3 and PML ChIP-seq (mm9, chr1: 132,461,098 – 139,182,269). **G–I** Zoom of promoter regions showing H3.3 and PML ChIP-seq **G** Nr5a2; mm9; chr1:138,666,969–138,977,687, **H** Etnk2; mm9; chr1:135,155,366–135,381,931, and **I** Lrrn2; mm9; chr1:134,680,403–135,017,359

We and others have previously shown that PML is essential for regulating deposition of histone H3.3 at telomeres and other DNA repeats [33, 34, 37, 38], but it was unclear if this association extended to other genomic regions. Consistent with previous findings of strong associations between PML-NBs and histone H3.3, immunofluorescent (IF) staining showed clear localization of DAXX/ATRX chaperone complex at PML-NBs in mouse embryonic stem (ES) cells (Fig. 1A). We next performed ChIP-sequencing of histone H3.3 and PML in mouse ES cells to identify the genomic sites where these proteins co-localized. In agreement with previous studies [33, 34], we detected enrichment of H3.3 and PML at telomeres by ChIP relative to input sequencing (Fig. 1B). The binding of H3.3 and PML at other DNA repeats are also shown as controls (Additional file 1: Fig. S1A); both H3.3 and PML are not enriched at these repeats with the exception of the presence of H3.3 at the IAPEz elements. When mapped across the genome, we found that H3.3 and PML localized predominantly to promoters and did not co-localize at other genomic sites (Fig. 1C; Additional file 1: Fig. S1B-C). In addition, H3.3 showed a smaller peak of enrichment at the transcription terminus (Fig. 1C) and was also enriched at enhancers while PML is only present at low levels (Additional file 1: Fig. S1D, E). Given H3.3 and PML localize to promoters, we analysed ChIP enrichment at individual promoters. ChIP-seq read density heatmaps and scatterplot of ChIP-reads show a high degree of correlation between H3.3 and PML at promoters (Fig. 1D, E). Overall, we observed a general concordance between H3.3 and PML distribution across the genome (Fig. 1F) particularly at promoters (Fig. 1G–I), which is not true for unrelated modifications (e.g., H3.3 and H3K27me3, Additional file 1: Fig. S1F, G), demonstrating that H3.3 and PML are closely associated and bind chromatin at similar locations.

### Pediatric glioma H3.3 (K27M/G34R) point mutations disrupt PML-NBs

Specific point mutations in H3.3 (K27M/G34R) and its ATRX chaperone are frequent events in pediatric gliomas [19]. Given H3.3/ATRX co-localize with PML (Fig. 1A), and PML-NBs are a known oncogenic driver in acute promyelocytic leukemia, we asked if the H3.3/ATRX mutations might be affecting the PML pathway in pediatric gliomas. To assess this possibility, we used three genetically modified mouse ES cell lines—H3.3 K27M, H3.3 G34R, and an H3.3 G34R/ATRX KO double mutant (DM) that recapitulates the mutation combination that is frequently observed in pediatric gliomas [19]. The H3.3 K27M (Additional file 1: Fig. S2A, B) and G34R [29] mutations were targeted to *H3f3a* and have been previously described [29, 53].

**Fig. 2:**
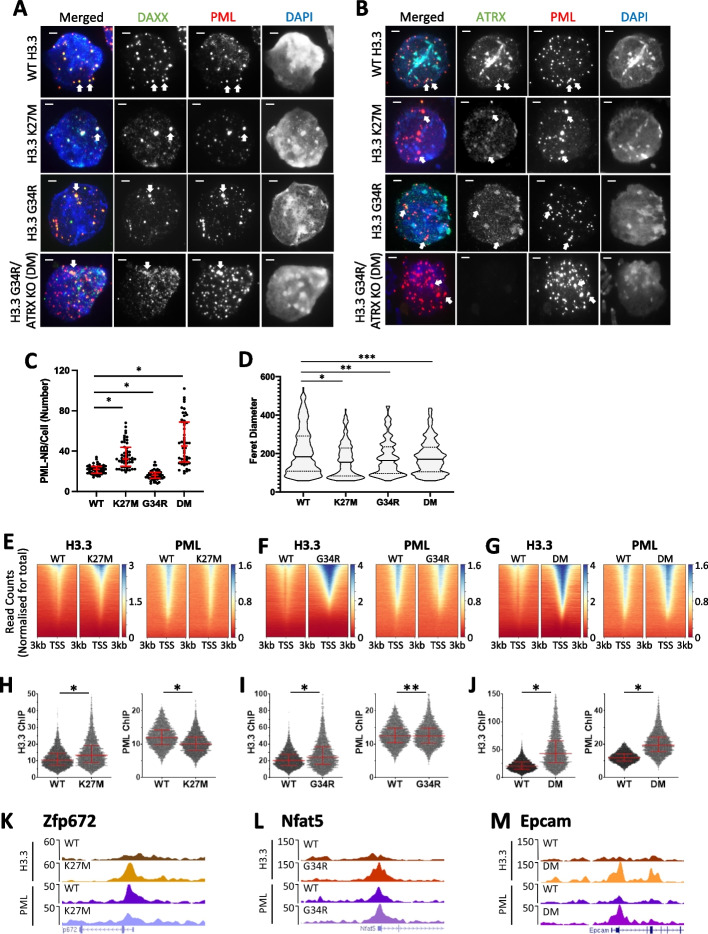
PML and H3.3 profiles in mouse ES cells with single-copy H3.3 (K27M/G34R) point mutations, and ATRX KO mutations. Immunofluorescence of **A** PML (red) and DAXX (green) and **B** PML (red) and ATRX (green), in WT, H3.3 K27M, H3.3 G34R, and H3.3 G34R/ATRX KO (double mutant; DM) mouse ES cell lines. Scale bar 2 µM.** C** Quantitation of PML-NBs per cell. Fifty nuclei (*n* = 50) were counted from three independent immunofluorescence experiments per cell line. Dots represent counts per cell (WT; H3.3 K27M; H3.3 G34R; DM). *P*-values were calculated using two-tailed Student’s *t* test (*P < 0.0001). **D** dSTORM quantitation of Feret diameter (size) of PML-NBs dies in WT (*n* = 292), H3.3 K27M (*n* = 200), H3.3 G34R (*n* = 276), and H3.3 G34R/ATRX KO (DM) (*n* = 289) cells. 25th, median, and 75th percentiles are shown. *P*-values were calculated using a two-tailed Mann–Whitney *U* test (*P < 0.0005, **P < 0.01, **P < 0.05). **E–G** ChIP-seq read count density heatmaps (normalized for total read counts) of H3.3 (left panel) and PML (right panel) ChIP-seq at transcriptional start sites (TSS) ± 3 kb, sorted by H3.3 enrichment in **E** H3.3 K27M, **F** H3.3 G34R, and **G** DM mutant cell lines, compared to WT cells. **H,I** H3.3 (left panel) and PML (right panel) ChIP-seq read counts at H3K4me3 enriched promoters (TSS ± 1 kb) in WT and **H** H3.3 K27M,** I** H3.3 G34R, and **J** DM cell lines. *P*-values were calculated using two-tailed Student’s *t* test (*P < 0.0001, **P < 0.0005). 25th, median, and 75th percentiles are shown.** K–M** Representative UCSC genome browser profiles of H3.3 and PML ChIP-seq in WT, **K** H3.3 K27M (Zfp672; mm9; chr11:58,105,009 – 58,165,027), **L** H3.3 G34R (Nfat5; mm9; chr8:109,772,915 – 109,862,824), and **M** DM cell lines (Epcam1; mm9; chr17:87,986,869–88,086,768)

Using a combination of IF and ChIP-seq, we found severe disruptions in the nuclear and genomic distribution of PML and H3.3 in all three mutant cell lines (H3.3 K27M, H3.3 G34R, and H3.3 G34R/ATRX KO [DM]). Compared to the WT mouse ES cells, the three mutant cell lines showed altered PML-NB numbers and size distribution profiles (Fig. 2A–D; Additional file 1: Fig. S2C). These alterations in PML-NBs in the mutant cell lines were not caused by reduced PML expression (Additional file 1: Fig. S2D). The H3.3 K27M mutant line showed increased number of PML-NBs per cell from a median of 22 in WT cells to a median of 32 (*n* = 50, *P* < 0.0001) (Fig. 2C), but the PML-NBs were smaller in size (*P* < 0.0005) (Fig. 2A, B, D). The H3.3 G34R mutant showed a decrease in the number of PML-NBs (median = 16, *n* = 50,* P* < 0.0001) (Fig. 2C), and the PML-NBs were also smaller in size (*P* < 0.01) (Fig. 2A, B, D). The addition of an ATRX mutation in H3.3 G34R mutant (DM) increased the number of PML-NBs by 2.5-fold relative to WT (median = 45, *n* = 50, *P* < 0.0001) (Fig. 2C), and altered the size distribution profile of PML-NBs (*P* < 0.05) (Fig. 2A, B, D).

In order to accurately assess the increased heterogeneity of PML-NB sizes in the mutant lines (Fig. 2A, B, D), we performed super-resolution imaging of PML-NBs using the single molecule-based dSTORM method (Fig. 2D; Additional file 1: Fig. S2C). In general, the H3.3 K27M and H3.3 G34R mutations decreased the median average Feret diameter of PML-NBs from 182 to 154 nm (*P* < 0.0001) and 163 nm respectively (*P* < 0.01), with a skew towards smaller PML-NBs in both mutants (Fig. 2D Additional file 1: Fig. S2C), demonstrating that H3.3 point mutations severely disrupt the formation of PML-NBs. These alterations in PML-NBs in the histone H3.3 mutants were not caused by the changes in the PML protein expression (Additional file 1: Fig. S2D), and notably, they were accompanied by changes in DAXX and ATRX distribution (Fig. 2A, B). Interestingly, the combination of H3.3 G34R/ATRX KO (DM) resulted in a reduced spread of size distribution (WT interquartile range = 183, DM IQR = 126, *P* < 0.05) (Fig. 2D), suggesting that there is a complex interplay between H3.3 mutations, PML-NB and the ATRX/DAXX chaperone complex.

To better assess specific differences in distribution profiles, we analyzed ChIP-seq of H3.3 and PML at promoters in the three mutated cell lines. Unexpectedly, we found that all three cell lines (H3.3 K27M, H3.3 G34R, H3.3 G34R/ATRX KO [DM]) showed significantly increased H3.3 incorporation at promoters (Fig. 2E–M). Notably, H3.3K27M and H3.3G34R consistently show an accumulation pattern distinct from that of WT H3.3. Specifically, they consistently display an enrichment pattern over the transcriptional start sites (TSS), whereas WT H3.3 consistently demonstrates a split enrichment pattern around the nucleosome-free region. Density heatmaps of H3.3 and PML ChIP-seq reads centered on TSS (TSS ± 3 kb) (*n* = 23,629) and sorted by H3.3 enrichment in K27M and G34R mutant cells, showed increased H3.3 concomitant with decreased PML (Fig. 2E, F). DM cells showed increased H3.3 and PML at all promoters, and increased PML correlated with increased H3.3 (Fig. 2G). In addition, the H3.3 mutations also appear to disrupt the H3.3 distribution profile at TSS. ChIP-seq of H3.3 in WT cells shows nucleosome depleted regions at TSS which is lost in the H3.3 mutants, which may indicate high levels of H3.3 at promoters or rapid exchange of H3.3 nucleosomes linked to RNA Pol II transcription at these sites.

As active promoters were enriched for PML and H3.3, we quantitated H3.3 and PML ChIP-seq reads at promoters (TSS ± 1 kb) which were enriched for H3K4me3 as a mark of promoter activity (*n* = 5910). We observed an average increase in the median counts of H3.3 (WT = 10.4, K27M = 13.3, *P* < 0.0001) and decreased PML in the H3.3 K27M mutant relative to WT (WT = 11.9, K27M = 10, *P* < 0.0001) (Fig. 2H). The H3.3 G34R mutation also caused increased median H3.3 (WT = 20.4, G34R = 24.4, *P* < 0.0001) but did not alter the overall median of PML (WT = 11.9, G34R = 11.9) although significant disruptions were observed at individual promoters (paired Student’s *t* test *P* < 0.0005) (Fig. 2I). The H3.3 G34R/ATRX KO (DM) mutations acted in combination to significantly increase H3.3 (WT = 11.0, DM = 43.1, *P* < 0.0001) and PML (WT = 11.9, DM = 19.4, *P* < 0.0001) at active promoters (Fig. 2J). Representative H3.3 and PML ChIP-seq profiles are shown for each mutant (Fig. 2K–M).

Taken together, these results suggest a complex interplay between H3.3 mutations, ATRX, and PML. Although there is not a strict relationship between onco-histone H3.3 and PML occupancy, it is clear that H3.3 mutations affect the formation of PML-NBs and the localization of PML. The exact effects appear to vary depending on the specific mutation (K27M/G34R) and the presence of ATRX. Nonetheless, all the tested mutations resulted in increased H3.3 incorporation at promoters which could trigger a self-reinforcing feedback loop where disruption of PML leads to increased incorporation of mutant H3.3.

### H3.3 K27M mutations disrupt PML-NBs and H3.3 incorporation in diffuse midline gliomas

**Fig. 3:**
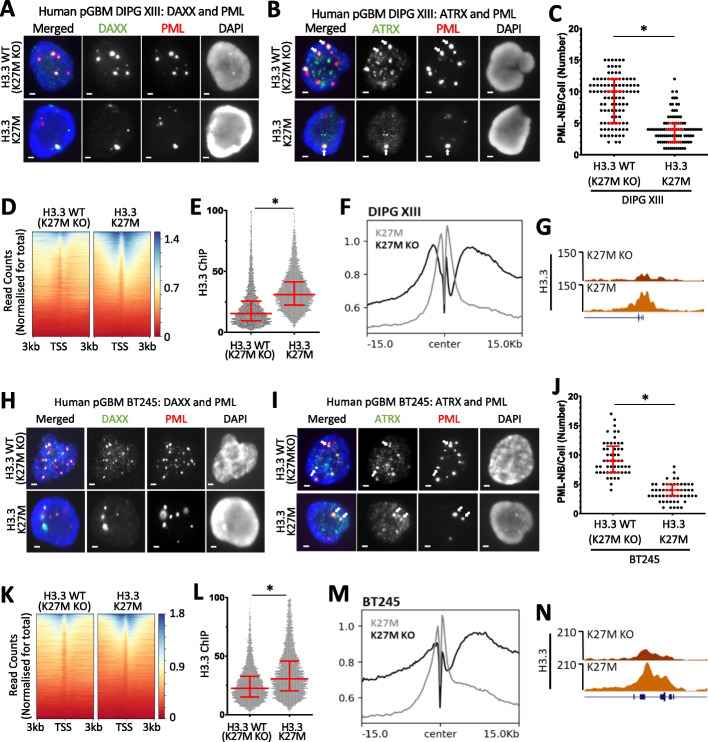
PML and H3.3 profiles in H3.3 K27M mutant patient-derived glioma cells. Immunofluorescence of **A** PML (red) and DAXX (green) and **B** PML (red) and ATRX (green), in H3.3 K27M in the DIPG XIII patient-derived glioma cell line compared to an H3.3 WT (K27M KO) isogenic control. Scale bar 2 µM. **C** PML-NBs per cell in H3.3 K27M and H3.3 WT (K27M KO) DIPG XIII cells (*n* = 103). *P*-values were calculated using two-tailed Student’s *t* test (*P < 0.0001). 25th, median, and 75th percentiles are shown. **D** ChIP-seq read count density heatmaps (normalized for total read counts) of H3.3 ChIP-seq at TSS ± 3 kb in H3.3 WT (K27M KO) and H3.3 K27M DIPG XIII cells. **E** ChIP-seq read counts at H3K27ac enriched promoters (TSS ± 1 kb) in H3.3 WT (K27M KO) and H3.3 K27M DIPG XIII cells (*n* = 5108). *P*-values were calculated using two-tailed Student’s *t* test (*P < 0.0001). 25th, median, and 75th percentiles are shown. **F** Composite profile of H3.3 at H3K27ac enriched promoters, centered on transcriptional start sites in H3.3 K27M DIPG XIII glioma cells compared to H3.3 WT (K27M KO) isogenic control. **G** Representative UCSC genome browser profile of H3.3 ChIP-seq in H3.3 K27M DIPG XIII and H3.3 WT (K27M KO) isogenic control (Ak3; hg38; chr9:4,635,308 – 4,827,283). **H–N** Experiments repeated across an independent (BT245) H3.3 K27M patient-derived glioma cell line. **H** Immunofluorescence of PML (red) and DAXX (green) and **I** PML (red) and ATRX (green), in H3.3 K27M BT245 cells compared to an H3.3 WT (K27M KO) isogenic control. Scale bar 2 µM. **J** PML-NBs per cell in H3.3 K27M BT245 and H3.3 WT (K27M KO) cells (*n* = 53). *P*-values were calculated using two-tailed Student’s *t* test (*P < 0.0001). **K** ChIP-seq read count density heatmaps of H3.3 ChIP-seq at TSS ± 3 kb in H3.3 WT (K27M KO) and H3.3 K27M BT245 cells. **L** ChIP-seq read counts at H3K27ac enriched promoters (TSS ± 1 kb) in H3.3 WT (K27M KO) and H3.3 K27M BT245 cells. *P*-values were calculated using two-tailed Student’s *t* test (*P < 0.0001). 25th, median, and 75th percentiles are shown.** M** Composite profile of H3.3 at promoters, centered on transcriptional start sites in H3.3 K27M BT245 ells compared to H3.3 WT (K27M KO) isogenic control.** N** Representative UCSC genome browser profile of H3.3 ChIP-seq in H3.3 K27M BT245 and H3.3 WT (K27M KO) isogenic control. Rims2; hg38; chr8:103,455,749 – 103,545,748

To determine if H3.3 mutations alter PML-NBs in pediatric gliomas, we assessed PML and H3.3 in two H3.3 K27M mutated patient-derived glioma cell lines (DIPG XIII, BT245). Isogenic cells where the H3.3 K27M mutated allele was deleted [termed as “H3.3 WT (K27M KO)”] leaving 3 copies of WT H3.3 and all other mutations [20, 24], were used as controls (Additional file 1: Fig. S3). Despite the complex mutational background of these cells, a single copy of H3.3 K27M was sufficient to cause a marked decrease in PML-NBs in the H3.3 K27M glioma cells compared to the H3.3 WT (K27M KO) controls (Fig. 3A–C). The H3.3 WT (K27M KO) controls had a median average of 10 PML-NBs per cell compared to a median average of 4 in the H3.3 K27M mutant line (*n* = 103, *P* < 0.0001) (Fig. 3C). Density heatmaps of H3.3 ChIP-seq reads centered on transcriptional start sites (TSS ± 3 kb) (*n* = 20,534) showed increased H3.3 in K27M mutated DIPG XIII patient-derived glioma cells relative to the H3.3 WT (K27M KO) control (Fig. 3D), similar to the mouse ES cells heterozygous for H3.3 K27M and G34R (Fig. 2). Quantitation of H3.3 ChIP-seq reads at active promoters (TSS ± 1 kb), as determined by H3K27ac enrichment (*n* = 5108), reinforced this finding with increased median read count average from 22.5 in H3.3 WT (K27M KO) cells to 30.6 in the H3.3 K27M cells (*P* < 0.0001) (Fig. 3E). A composite profile of all genes centered on the TSS and a representative promoter profile are also shown (Fig. 3F, G). These results were reproduced in a second (BT245) patient-derived glioma line (Fig. 3H–N). The number of PML-NBs were reduced from a median average of 9 in H3.3 WT (K27M KO) cells to 4 in the H3.3 K27M mutant (*n* = 53, *P* < 0.0001) (Fig. 3J). Read counts of H3.3 at active promoters increased from a median of 22.5 to 30.6 in H3.3 WT (K27MKO) cells compared to H3.3 K27M mutants (*n* = 5197, *P* < 0.0001) (Fig. 3L). These findings, together with the results in heterozygous mutant mouse ES cells (Fig. 2), suggest that the H3.3 K27M mutation is sufficient to cause severe disruptions in PML-NBs (Fig. 3A–C, H–J) while simultaneously increasing the incorporation of mutant histone H3.3 at promoters (Fig. 3D–G, K–N).

### PML-NB disruption is an H3.3-specific phenotype

**Fig. 4:**
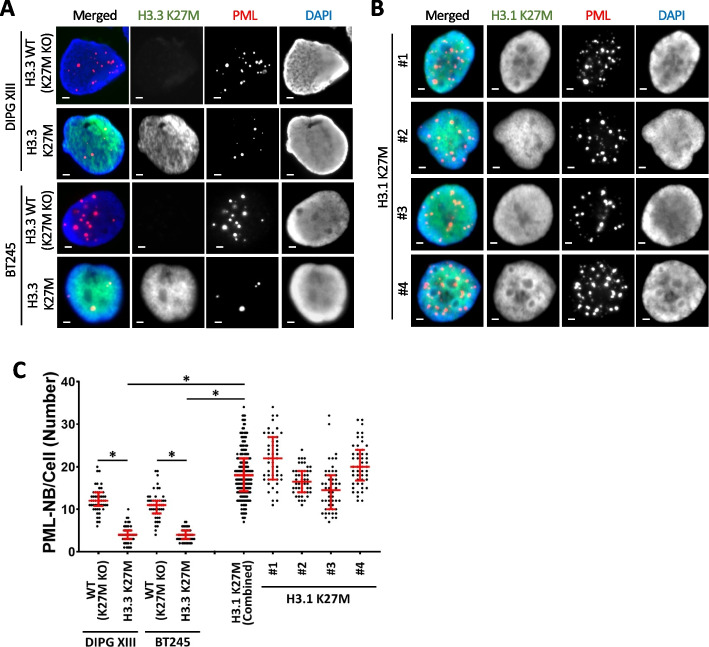
PML nuclear bodies in H3.3 K27M vs canonical H3.1 K27M mutated patient-derived glioma cells. Immunofluorescence of PML (red) and H3K27M (green) in **A** DIPG XIII and BT245 H3.3 K27M mutated patient-derived glioma cells compared to isogenic H3.3 WT (K27M KO) controls. **B** H3.1 K27M mutated patient-derived glioma cells: #1 (SU_DIPG_36), #2 (ICR_B184_2D), #3 (SU_DIPG_33), and #4 (SU_DIPG_4). Scale bar 2 µM. **C** Quantitation of PML-NBs per cell. 50 nuclei (*n* = 50) were counted from three independent immunofluorescence experiments per cell line. Dots represent counts per cell (WT (K27MKO); H3.3 K27M in DIPG XIII and BT245 cells; #1; #2; #3; #4 H3.1 K27M lines). 25th, median, and 75th percentiles are shown. H3.1 K27M (combined) shows an aggregate of all four H3.1 K27M cell lines. *P*-values were calculated using two-tailed Student’s *t* test (*P < 0.0001)

Although there are fewer copies of histone H3.3 than canonical histone H3.1/2 genes (2 vs 13 copies) [5], and H3.3 represents a minor fraction of total H3 [18], H3.3 is far more frequently mutated in pediatric gliomas (100% G34R, 83% K27M) [19]. This is strongly indicative of an oncogenic mechanism which is specific to the H3.3 variant. PML disruption is an excellent H3.3-specific mechanistic candidate as this is a known oncogenic driver which also interacts with H3.3. To determine if PML disruptions are indeed specific to H3.3 mutations, we compared H3.3 K27M with four H3.1 K27M mutated patient-derived glioma cell lines SU_DIPG_36 (#1), ICR_B184_2D (#2), SU_DIPG_33 (#3) and SU_DIPG_4 (#4). The presence of K27M mutations were confirmed by Western blots with an H3K27M antibody (Additional file 1: Fig. S4A). Immunohistochemistry staining for PML in H3.1 K27M mutants showed punctate and numerous PML-NBs similar to H3.3 WT (K27M KO) cells. While both H3.3 K27M mutants (DIPG XIII median = 4; BT245 median = 4, *n* = 50) had reduced numbers of PML-NBs relative to WT H3.3 (K27M KO) (DIPG XIII median = 14, BT245 median = 11, *n* = 50, *P* < 0.0001) (Fig. 4A, C; Additional file 1: Fig. S4B), the H3.1 K27M mutants had higher numbers of PML-NBs on average (median #1-#4 = 22; 16.5; 14.5; 20; *n* = 50; H3.3 K27M aggregate median = 22, *n* = 200, *P* < 0.0001) compared to the WT H3.3 (K27M KO) lines (Fig. 4B, C; Additional file 1: Fig. S4B). As there are no genuine “WT” representative of these cells, it is not possible definitively determine if the H3.1 mutations truly increase PML-NBs relative to a true WT population. It is nonetheless clear that H3.3 and H3.1 K27M mutations have distinct effects on PML-NBs. The loss of PML-NBs is an H3.3-specific phenomenon and suggests that PML disruptions can contribute to oncogenesis in H3.3-mutated pediatric gliomas.

### Knockdown of PML impairs glial differentiation similar to H3.3 K27M mutations

**Fig. 5:**
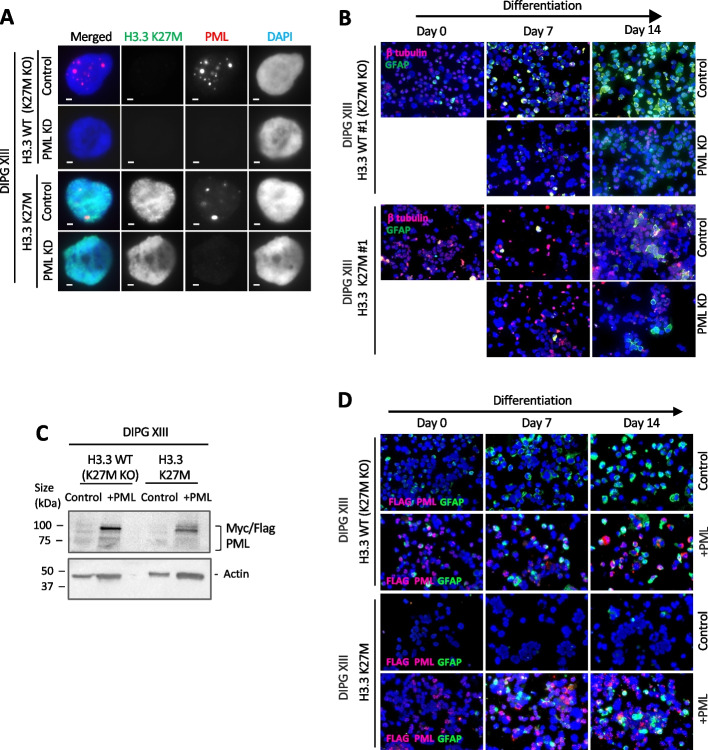
PML knockdown inhibits differentiation in H3.3 WT (K27M KO) glioma cells. **A** Immunofluorescence of K27M (green) and PML (red) in DIPG XIII H3.3 WT (K27M KO) and H3.3 K27M pediatric glioma cells (clone #1) with and without doxycycline-induced PML knockdown. Scale bar 2 µM.** B** Immunofluorescence staining of GFAP glial marker (green) and β-tubulin (red) of DIPG XIII H3.3 WT (K27M KO) and H3.3 K27M glioma cells (clone #1) with and without PML knockdown, over 7 and 14 days of culture in differentiation media. **C** Western blots of DIPG XIII H3.3 WT (K27M KO) and H3.3 K27M glioma cells overexpressing myc/flag tagged PML (denoted + PML) using anti-myc and actin antibodies. **D** Immunofluorescence staining of GFAP glial marker (green) and β-tubulin (red) of DIPG XIII H3.3 WT (K27M KO) and H3.3 K27M glioma cells with and without PML overexpression, over 7 and 14 days of culture in differentiation media

Previous studies have shown that H3.3 K27M inhibits glial differentiation [52, 57], a phenotype which is reminiscent of PML-mutated promyelocytic leukemias [51]. As H3.3 K27M also disrupts PML-NBs, we hypothesized that abnormal PML-NBs may be contributing to the failure in differentiation. To test this, we created CRISPR inducible (CRISPRi) PML-knockdown mutants in H3.3 K27M and H3.3 WT (K27M KO) patient-derived glioma lines (Fig. 5A) with a doxycycline inducible sgRNA and nuclease-deficient Cas9-KRAB CRISPR repressor system (Additional file 1: Fig. S5A, B). Doxycycline was added to induce knockdown of PML leading to the loss of PML-NB (Additional file 1: Fig. S5B) and we assessed differentiation by assaying a glial marker, GFAP. The H3.3 WT (K27M KO) cells showed a graded increase in GFAP staining over 7 and 14 days of growth in differentiation media [52, 57] (Fig. 5B; Additional file 1: Fig. S5C).

In agreement with previous studies, the H3.3 K27M mutant lines were unable to differentiate as determined by GFAP staining after 14 days (Fig. 5B; Additional file 1: Fig. S5 C), regardless of PML status. However, knockdown of PML inhibited differentiation in the H3.3 WT (K27M KO) cells, as evident from a substantially reduced GFAP staining compared to WT PML counterparts (Fig. 5B; Additional file 1: Fig. S5C). Consistent with the idea that PML-NBs are important for differentiation, exogenous overexpression of PML was able to partially rescue the differentiation block in H3.3 K27M cells. H3.3 K27M and K27M KO cells were transduced with lentiviral vectors expressing PML from a strong CMV promoter and induced to differentiate (Fig. 5C,D). A subset of H3.3 K27M cells overexpressing PML stained positive for GFAP after 14 days of differentiation (Fig. 5D), showing that overexpression of PML can partially compensate for differentiation deficits in H3.3 K27M mutants. Taken together, these results support the idea that H3.3 K27M-mediated disruption of PML-NB contributes to the failure to differentiate in pediatric gliomas.

### H3.3 K27M patient-derived glioma cells are sensitive to arsenic trioxide

PML-RARα mutations are known to cause differentiation arrest in acute promyelocytic leukemia. Treatment with arsenic trioxide (ATO) can overcome this differentiation block [58, 59] by first promoting PML-NB re-formation [60] and subsequent degradation [61] to facilitate clearance of leukemia cells [62, 63]. Our results strongly suggest that H3.3 K27M acts in an analogous manner and blocks differentiation by disrupting PML-NBs, meaning that ATO may also be effective at overriding these PML-NB defects and promote cell death in H3.3-mutated gliomas.

**Fig. 6:**
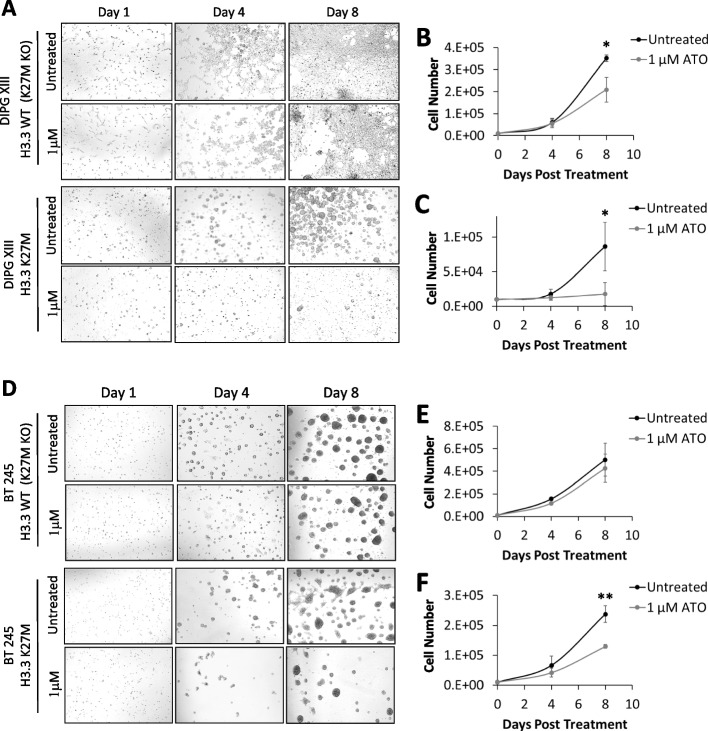
Arsenic trioxide treatment of H3.3 K27M mutated patient-derived glioma cells. **A** Phase-contrast microscopy of DIPG XIII H3.3 WT (K27M KO) and H3.3 K27M pediatric glioma cells treated with 1 µM of arsenic trioxide for 1, 4, and 8 days. Untreated cells are shown as controls. Cell counts of **B** H3.3 WT (K27M KO) and **C** H3.3 K27M pediatric glioma cells treated with 1 µM of arsenic trioxide for 0, 4, and 8 days compared to untreated controls. **D–F** Experiments were repeated in an independent BT245 H3.3 WT (K27M KO) and H3.3 K27M pediatric glioma cell line. Points and error bars represent the mean average and standard deviation of three independent experiments. *P*-values were calculated using two-tailed Student’s *t* test (*P < 0.05 **P < 0.005)

To test this, we first assessed the effects of ATO treatment on PML protein expression in pediatric glioma cell lines. We found that glioma cells treated with ATO behaved similarly to PML-RARα mutated APL. Both the H3.3 K27M mutant and the corresponding H3.3 WT K27M KO control line showed increased PML at 4 h post-treatment with ATO, not only in the soluble (S3) fraction but also in the insoluble pellet (P) fraction (Additional file 1: Fig. S6A, B). This increase of PML insolubility is likely triggered by the hyper-sumoylation of PML, as indicated by the presence of high molecular weight PML isoforms, following ATO treatment [60, 61, 64]. This was then followed by a decline in PML levels at the 24-h timepoint (Additional file 1: Fig. S6A, B), due to degradation facilitated by the proteasome machinery, similar to reports for PML-RARα APL [60, 61, 64]. In line with these changes, we observed increased PML-NBs by immunostaining at 1 and 4 h followed by a reduction at 24 h post-treatment with ATO (Additional file 1: Fig. S6 C, D). We next tested sensitivity of H3.3-mutated gliomas to ATO by treating H3.3 K27M pediatric glioma cell lines and H3.3 WT (K27M KO) controls with 1 µM ATO for 8 days. Treatment of K27M KO cells with 1 µM ATO resulted in a moderate decrease in mean cell numbers relative to untreated controls (Fig. 6A, B). In comparison, the H3.3 K27M mutant counterparts were much more sensitive to treatment with ATO and reduced cell numbers by 80% relative to untreated controls after 8 days (*P* < 0.05) (Fig. 6A, C). These findings were reproduced in a second glioma line (BT245) which was also hypersensitive to ATO (Fig. 6D–F). BT245 H3.3 WT (K27M KO) cells were largely unaffected by ATO treatment (15% decrease mean average, not significant), while ATO reduced cell numbers by an average of 45% in the H3.3 K27M mutants (*P* < 0.005). Of note, three additional H3.3 K27M mutant lines, which showed low number of PML-NBs (Additional file 1: Fig. S6E), also showed significant sensitivity to ATO treatment (Additional file 1: Fig. S6F).

Importantly, exogenous overexpression of PML reduced sensitivity to ATO in H3.3 K27M cells, while increasing sensitivity in WT H3.3 (K27M KO) controls. Histone-mutated glioma cells were transduced with lentiviral CMV-PML and treated with 1 µM ATO for 8 days. Overexpression of PML in WT H3.3 (K27M KO) cells increased sensitivity to ATO and there was a marked decrease in cell viability when cells were treated with ATO (Additional file 1: Fig. S6G). In contrast, overexpression of PML in H3.3 K27M glioma cells reduced overall cell viability by 30% relative to untreated controls; however, treatment with ATO had no further effects on cell viability, and CMV-PML cells were resistant to ATO compared to WT PML counterparts (Additional file 1: Fig. S6H). These results altogether demonstrate that H3.3 K27M-mutated glioma cells are hypersensitive to ATO treatment in culture. As overexpression of PML partially reduces this sensitivity, it is likely that ATO sensitivity is mediated through disruptions of PML in H3.3 K27M cells.

### The IDH1 R132H mutations found in glial and myeloid malignancies are disruptive to PML-NBs

Our results indicate that PML is a common link between H3.3 mutations in pediatric gliomas and PML-mutated promyelocytic leukemias. Interestingly, glial and myeloid malignancies are both also frequently mutated in IDH1 and IDH2 [65]. Substitution point mutations in IDH1 (R132H) and IDH2 (R172K; R140Q) are common events in low-grade (II/III) glioma (> 75%) [66] and acute myeloid leukemias (AML) (20%) [67], but rare in other cancers [65]. We have previously shown that the IDH1 R132H mutation acts in the same pathway as the H3.3 G34R mutation in inhibiting KDM4B H3K9/K36 demethylase and promoting ALT telomere maintenance pathway in ATRX mutated cancers [53], a phenotype that necessitates the formation of ALT-associated PML bodies. Furthermore, the ATO/ATRA drug combination which targets PML-NBs in acute promyelocytic leukemias has been shown to be effective in IDH-mutated AML [68], suggesting a possible PML involvement.

**Fig. 7:**
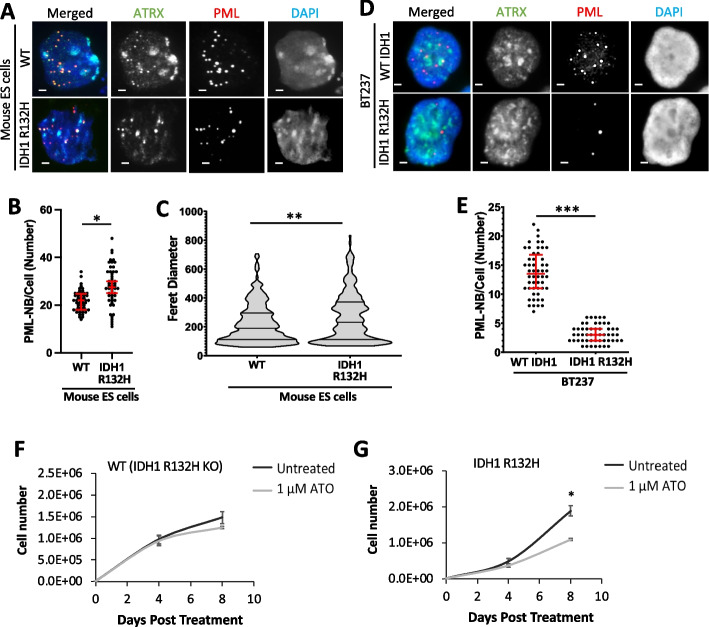
PML-NBs and arsenic trioxide treatment of IDH1 R132H mutated oligodendroglioma cells.** A** Immunostaining of ATRX (green) and PML (red) in WT and heterozygous IDH1 R132H mouse ES cells. Scale bar 2 µM. **B** Quantitation of PML-NBs per cell. Fifty nuclei (*n* = 50) were counted per cell line from three independent immunofluorescence experiments. Dots represent counts per cell. *P*-values were calculated using two-tailed Student’s *t* test (*P < 0.0001). **C** dSTORM quantitation of Feret diameter (size) of PML nuclear bodies in WT (*n* = 292), IDH1 R132H (*n* = 256) cells. 25th, median, and 75th percentiles are shown. *P*-values were calculated using a two-tailed Mann–Whitney *U* test (**P < 0.005). **D** Immunostaining of ATRX (green) and PML (red) in IDH1 WT (R132H KO) and BT237 IDH1 R132H patient-derived glioma cells. Scale bar 2 µM. **E** PML-NBs per cell in IDH1 KO and IDH1 R132H mutant cells. Dots represent counts per cell (*n* = 103). 25th, median, and 75th percentiles are shown. *P*-values were calculated using two-tailed Student’s *t* test (***P < 0.0001). Cell counts of **F** IDH1 WT (R132H KO) and **G** IDH1 R132H pediatric glioma cells treated with 1 µM of arsenic trioxide for 0, 4, and 8 days compared to untreated controls. Points and error bars represent the mean average and standard deviation of three independent experiments. *P*-values were calculated using two-tailed Student’s *t* test (*P < 0.005)

We therefore stained for PML-NBs in mouse ES cells with a heterozygous IDH1 R132H substitution mutation [53]. Much like the H3.3 point mutations (Fig. 2), the IDH1 R132H mutation disrupted both the number and size of PML-NBs (Fig. 7A–C; Additional file 1: Fig. S7A). The median average number of PML-NBs per cell increased from 22 in WT cells (*n* = 50) to 28 PML-NBs per cell in the IDH R132H mutant (*n* = 50, *P* < 0.05) (Fig. 7A, B). We also observed heterogeneity of PML-NB Feret diameter in IDH R132H mutants (*P* < 0.005), with a median average increase from 182 nm in WT cells (*n* = 292) to 231 nm in IDH R132H mutants (*n* = 259) (Fig. 7A, C; Additional file 1: Fig. S7A), but this PML-NB alteration was not caused by a significant change in PML protein levels (Additional file 1: Fig. S7B).

A similar result was obtained in an IDH1 R132H mutated patient-derived BT237 oligodendroglioma cell line [69]. Like the H3.3 K27M mutated gliomas (Fig. 3), IDH1 R132H mutated cells showed a marked decrease in PML-NBs but also lower levels of PML protein relative to an isogenic IDH1 WT (R132H KO) control where the R132H mutated allele was deleted (Fig. 7D, E; Additional file 1: Fig. S7C, D). In addition, IDH1 R132H mutated cells were more than twice as sensitive to treatment with 1 µM of ATO compared to the WT counterparts. ATO treatment had no significant effects on IDH1 WT (R132H KO) cell numbers, while IDH1 R132H mutant cell numbers were reduced by 42% relative to untreated controls (*P* < 0.005) (Fig. 7F, G). Combined, these results demonstrate that disruption of the PML pathway is an oncogenic driver in pediatric gliomas and could potentially be targeted for therapy. Furthermore, we show that multiple mutations—H3.3 G34R, H3.3 K27M, IDH1 R132H—disrupt PML-NBs, and glial and myeloid malignancies are particularly prone to mutations in this pathway.

## Discussion

The discovery of histone H3.3 point mutations in pediatric gliomas were a major breakthrough in our understanding of these brain tumors [1, 2]. Additional H3.3 mutations have now been identified across a range of cancers including H3.3 G34W/L in giant cell tumor of bone [70], H3.3 K36M mutations in chondroblastomas [70], and H3.3 K27M/G34W/R mutations in osteosarcomas [71]. Numerous studies have detailed a diverse array of chromatin modifications which are disrupted in the presence of these mutations [3]. However, it has proven difficult to identify mechanisms which can satisfactorily account for the widespread genome alterations associated with these point mutations. It is also unclear why the histone variant H3.3 is frequently and disproportionately mutated in cancers relative to canonical H3.1/2 and, indeed, how these mutations promote oncogenesis. We have identified PML and related pathways as oncogenic drivers which are disrupted by histone H3.3 point mutations. Given that PML-NBs are involved in many genomic processes and PML mutations definitively drive oncogenesis in acute promyelocytic leuaemia, our findings could help address many of the open questions surrounding H3.3 mutations.

We found that the two H3.3 point mutations (G34R/K27M) which are common in pediatric brain cancers disrupted the formation of PML-NBs. Interestingly, although both H3.3 mutations alter the size and number of PML bodies relative to WT H3.3, the specific effects vary depending on the point mutation (G34R, K27M), the presence of additional mutations (e.g., ATRX), and the cell type (human glioma, mouse ES cells). As PML-NBs undergo a cyclical process of aggregation, maturation, and degradation [72–74], the variation in size and numbers of PML-NBs between mutants could be due to interference with different stages of this process. For example, the PML-RARα mutation appears to block the formation of normal PML-NBs as treatment with ATO first promotes the formation of functional PML-NBs, and subsequent degradation of hypersumoylated PML-NBs [60, 61, 64]. The H3.3/ATRX mutations could interfere with different points of the nucleation/maturation/degradation cycle. ATO appears to play a general role in promoting nucleation and degradation of PML-NBs and can therefore override these hurdles regardless of the phase or specific cause of PML-NB cycle arrest.

Although we are at present not able to account for the exact mechanisms behind PML disruptions, it is nonetheless clear that normal functional PML-NBs are required for differentiation, and H3.3 mutations interfere with this process. Considering the highly complex and dynamic interplay between H3.3, DAXX, ATRX, and PML-NBs, several reasons may account for this, but the most likely explanation is that the different H3.3 mutations may affect the binding of H3.3 interacting proteins. PML-NBs are dynamic structures that host a diverse array of constantly changing nuclear proteins, so that PML-NBs may have different compositions within a cell [39, 41, 42, 75]. The mutated residues on H3.3 could potentially interfere with a range of PML-associated proteins which promote or disrupt the nucleation or degradation of PML-NBs. If the mutated histones are acting through protein partners which reside in PML bodies, this may explain why it has been unexpectedly difficult to identify binding partners that are directly affected by H3.3 mutations in vivo. The insoluble nature of PML-NBs is an additional hurdle that can be overlooked with conventional biochemistry protocols. It will be very interesting to see if potential mutant H3.3 interacting partners can be identified using techniques which are optimized for extraction of PML-NBs.

One of the more puzzling aspects of these histone point mutations are the diverse and widespread chromatin alterations which have been observed in mutated cells. For example, H3.3 K27M has been reported to interfere with H3K27me3 [20–22], H3K27ac [23–25], H3K36me2 [26, 27], H3K4me3 [28], and DNA methylation [21], and a similar range of chromatin changes have been associated with the G34 mutations [22, 29–31]. The complex and disparate genomic alterations argue against a simple model where each mutation affects a single chromatin modifier (e.g., K27M inhibits PRC2) and suggests instead that a range of chromatin proteins are disrupted by H3.3 tail mutations. In addition to ATRX/DAXX/H3.3, PML-NBs are known to host a range of nuclear proteins, including chromatin modifiers such as SETDB1 [48], PRC2 [48], acetyltransferases, deacetylases, and DNA demethylases [39]. This arrangement places H3.3 in close proximity to many chromatin modifiers which might interact with, and be disrupted by, the mutant tail. This allows PML-NBs to amplify the effects of H3.3 mutations by concentrating and increasing stochastic interactions with binding partners. This PML model would allow for cascading effects on modifiers and modifications across the genome which could occur independently of H3.3 genomic incorporation sites.

This could also contribute to the differences observed between H3.1 K27M and H3.3 K27M mutations. While the K27M substitution very likely affects the same chromatin modifiers, H3.1 vs H3.3 mutations are associated with distinct chromatin profiles [76], secondary mutations, and age-of-onset [77–79]. We now report that the H3.1 K27M mutation does not affect PML-NBs in the same manner as H3.3 K27M mutations, further emphasizing the differences between these mutations, which needs to be considered in future investigations and clinical trials.

Chromatin and transcriptional states are the major determinants of cell identity, so disruptions to chromatin often interfere with differentiation. We identify PML as one factor which is involved in glial differentiation and find that this pathway is adversely affected by H3.3 point mutations. This idea is consistent with the function of PML-NBs in the genome (e.g., transcription, silencing, stemness, senescence) [39], and the differentiation arrest of PML-mutated promyelocytic leukemias. As H3.3 K27M gliomas are also arrested in differentiation and H3.3 K27M interferes with PML-NBs, it is highly likely this pathway contributes to differentiation failure in pediatric gliomas. Furthermore, arsenic trioxide targets PML-NBs very effectively in acute promyelocytic leukemia and we find that this agent is similarly effective against histone-mutated gliomas. Taken together, these results strongly suggest that the PML pathway is a major contributor to oncogenesis in histone-mutated pediatric gliomas, and this pathway could represent a novel therapeutic target.

Our results have revealed PML as a common factor connecting pediatric gliomas and promyelocytic leukemias. Interestingly, mutations in the citric acid cycle genes, isocitrate dehydrogenase (IDH1/2), are common events in low-grade gliomas [66] and acute myeloid leukemias [67]. Isocitrate dehydrogenase converts isocitrate to α-ketoglutarate (α-KG), also known as 2-oxoglutarate (2-OG), which is required for the activity of 2-OG-dependent dioxygenases such as the JmjC histone demethylases (e.g., KDM4B) and TET DNA demethylases. The IDH point mutations (IDH1 R132H, IDH2 R172K/R140Q) produce 2-hydroxyglutarate instead of α-KG (2-OG) [80], and this oncometabolite inhibits the 2-OG-dependent dioxygenases [81, 82].

We previously demonstrated that inhibition of KDM4B, either through H3.3 G34R or IDH1 R132H, facilitates the formation of ALT-associated PML bodies [53]. We now find that the IDH1 R132H mutation also disrupts the formation of PML-NBs in pediatric gliomas. This strongly suggests that PML disruption is a common factor linking glial and myeloid malignancies, uniting the PML/H3.3/IDH mutations that are common in these types of cancers. We further note that the closely related chondroblastomas/chondrosarcomas have frequent mutations in H3.3 K36M and IDH1/2 respectively, and we predict that PML disruption would also feature in these tumors.

## Conclusions

We have demonstrated that H3.3 mutations disrupt PML-NBs in pediatric gliomas and contribute to oncogenesis. Similar to PML-mutated acute promyelocytic leukemias, the abnormal PML-NBs can be targeted with arsenic trioxide in H3.3-mutant pediatric gliomas, and this finding could have clinical applications. Furthermore, we found that the IDH1/2 point mutations, common in both glial and myeloid malignancies, also disrupt PML-NBs demonstrating that a range of disparate mutations converge on PML.

## Methods

### Human and mouse ES cell culture

Mouse ES cells were cultured in Dulbecco’s modified Eagle’s medium (DMEM) supplemented with 12% heat-inactivated fetal bovine serum, 10^3^ units/ml leukemia inhibitory factor (Merck), 0.1 mM β-mercaptoethanol, non-essential amino acids, L-glutamine, and penicillin/streptomycin. Cells were maintained in 37 °C incubator under 5% CO_2_. HEK293 cells were cultured in DMEM supplemented with 10% heat-inactivated fetal calf serum, L-glutamine, and penicillin/streptomycin. Cells were maintained at 37 °C in 5% CO_2_ incubator.

### Human patient-derived glioma cell culture

Human patient-derived glioma cell lines DIPG XIII, BT245 [20, 24] were cultured in human NeuroCult NS-A Proliferation Media (Stemcell Technologies) with 0.002% heparin, 10 ng/ml bFGF, 20 ng/ml rhEGF, penicillin, and streptomycin. Human SU_DIPG_4, SU_DIPG_33, SU_DIPG_36 [83] and ICR_B184_2D and BT237 anaplastic oligodendroglioma cells [69] were cultured in Neurobasal-A/DMEM/F12 media (Gibco Thermo Fisher, cat no. 10888–022 and 11,330–032) with NEAA, 2 mM Glutamax, penicillin/streptomycin, 10 mM HEPES, 1 mM sodium pyruvate, B27 supplement, 20 ng/ml rhEGF, 20 ng/ml bFGF, 0.002% heparin, 10 ng/ml PDGF-AA, and 10 ng/ml PDGF-BB. Human DIPG XIII glioma cells were differentiated using DMEM differentiation media (Gibco) with 10% fetal bovine serum containing penicillin/streptomycin, as previously described [52].

### Generation of H3.3 K27M mouse ES cell line

The single-copy H3.3 K27M mutant ES cells lines were created using a previously described conditional allelic replacement strategy [29, 84]. The targeting vector was identical with the previous study [84], except the yellow fluorescent protein coding sequence was replaced with the H3.3 K27M coding sequence. On exposure of targeted cells to Cre-recombinase, the WT H3.3 minigene was excised, and the mutant K27M minigene was brought under control of the endogenous *H3f3a* promoter. Mutant clones were selected by restoration of G418 sensitivity—because the *neo* cassette is excised together with the WT minigene, and confirmed by Southern blot.

### Southern blotting

Southern blots were performed as previously described [29, 84]. Briefly, cells were lysed with 0.5 ml of lysis buffer (50 mM Tris–HCl pH 8.0, 100 mM NaCl, 100 mM EDTA, 1% SDS). DNA was precipitated with ammonium acetate and isopropanol and washed with 70% ethanol. DNA was resuspended in 50 μl of TE buffer. Genomic DNA was digested with a fivefold excess of restriction enzyme and 5 μg was loaded. Blots were performed with Hybond XL membranes according to the manufacturer’s protocols (GE Healthcare), except that the pre- and hybridization solution was 5 × SSPE, 5 × Denhardt’s solution, and 1% SDS. All gels were depurinated and washed twice with 0.2 × SSC and 0.5% SDS. PCR primers for probe synthesis are shown below: Probe1, primer #1, ACCA TGCT GGGC TCTT TAC, primer #2, AACT GTGC TAGG CACA GC, amplicon 295 bp; Probe2, primer #1, ACTA GAAA AACC GGCC AA, primer #2, GCAT ATAC GGAG TCAG GGA, amplicon 306 bp.

### qPCR for expression analysis

RNA was extracted using Promega SV Total RNA Isolation Kit. cDNA was then synthesized using the High-Capacity cDNA Reverse Transcription Kit according to the manufacturer’s instructions (Thermo Fisher Scientific). Twenty nanograms of cDNA was combined with 0.5 μM of primers and FastStart DNA Master SYBR Green (Roche) in a 10 μl reaction and the expression levels of target genes were quantitated using the LightCycler® (Roche). As an internal control, primers specific for GAPDH were used in real-time polymerase chain reaction (PCR) analysis. The comparative cycle threshold (CT) method was used for data analyses and relative fold difference was expressed as 2 − ΔΔCT. Primers for GAPDH and GFAP transcripts are shown in Table S1 (in Additional file 2).

### Immunofluorescence analysis

Cells were harvested and resuspended in a hypotonic solution of 0.075 M KCl before being cytospun onto glass slides. Cells were rinsed with KCM buffer (120 mM KCl, 20 mM NaCl, 10 mM Tris–HCl at pH 7.2, 0.5 mM ethylenediaminetetraacetic acid (EDTA) 0.1% v/v Triton X-100 and protease inhibitor) (5 min, RT), followed by permeabilization with extraction buffer containing 0.5% Triton X-100 in KCM (5 min, RT), and blocked with 1% BSA (vol/vol) in KCM. They were then incubated with the relevant primary and secondary antibodies (37 °C, 1 h). After each round of antibody incubation, slides were washed thrice in KCM buffer. Slides were then fixed with KCM buffer containing 4% formaldehyde and mounted in mounting medium (Vectashield). Images were taken with a Zeiss imager M2 fluorescence microscope linked and AxioCam MRm CCD camera system.

### GFAP immunofluorescence

H3.3 K27M mutant and H3.3 WT (K27M KO) glioma cells were cultured in either complete neuronal media or DMEM and harvested by treatment with Accutase. Harvested cells were resuspended in 1 × PBS and sedimented onto slides using a Cytospin machine. Slides were subsequently fixed in 4% PFA in 1 × PBS, washed three times using 1 × PBS, extracted in ice-cold 1 × PBS with 0.1% Triton X-100, and blocked in 1 × PBS with 2% BSA for 5 min. Following blocking, the anti-GFAP and anti-β-tubulin antibodies (Additional file 2: Table S2) were applied (37 °C, 1 h) (1:500). Slides were washed twice in PBS, and 0.1% Tween-20 in PBS, before the secondary antibody (1:1000) was applied (45 °C, RT). Slides were washed, fixed, and left to air dry. Slides were mounted with DAPI in Vectashield media. Images were taken with a Zeiss imager M2 fluorescence microscope and AxioCam MRm CCD camera system.

### Antibodies

All antibodies used are listed in Table S2 (in Additional file 2).

### Preparation of cell lysates for Western blot analysis

Cells were washed with 1 × PBS, followed by lysis in ice-cold modified RIPA buffer (10 mM Tris–HCl, pH 8.0, 140 mM NaCl, 1 mM EDTA, 0.5 mM EGTA, 1% Triton X-100, 0.1% Sodium Deoxycholate, 0.1% SDS, protease inhibitors, 1 mM 4-(2-aminoethyl) benzenesulfonyl fluoride hydrochloride (ABESF)) for 5 min on ice, followed by sonication for10 s and centrifugation at 12,000 × *g* with a bench top centrifuge for 5 min. The supernatant was collected and diluted ¼ with 4 × SDS PAGE buffer, boiled for 5 min, and chilled on ice. Samples were loaded on a 10% SDS PAGE followed by Western blot analysis using the relevant antibodies.

For extraction of PML proteins, cells were washed with PBS 1 × , and resuspended in cytoskeleton buffer (CSK: 10 mM PIPES pH 6.8, 100 mM NaCl, 1 mM EGTA, 300 mM Sucrose, 3 mM MgCl_2_) supplemented with 1 mM DTT, 0.5% Triton X-100, protease Inhibitors and 1 mM ABESF [85]. After 5 min incubation on ice, the lysate was subjected to centrifugation at 900 × *g* for 3 min, and the supernatant was collected and labelled as S1 fraction. The pellet was washed with an additional volume of cytoskeleton buffer. Chromatin from the pellet was solubilized by DNA digestion with 10U of RNase–free DNase (Turbo DNAse; Invitrogen M610A) in CSK buffer for 20 min at 37 °C. Samples were pelleted at 2350 × *g* for 3 min at 4 °C and the supernatant was labelled as S2 fraction. After a wash in CSK buffer, the pellet was further extracted with 2 M NaCl in CSK buffer for 5 min at 4 °C, centrifuged at 2350 × *g* 3 min at 4 °C and the supernatant was labelled as S3 fraction. Remaining pellet was resuspended in 2 × PAGE buffer, sonicated for 10 s and boiled for 5 min (P). All the different fractions including S1, S1, S3, and P were diluted ¼ in 4 × PAGE buffer, boiled for 5 min. Samples were loaded on a 10% SDS PAGE followed by Western blot analysis using the relevant antibodies.

### Chromatin immunoprecipitation

Five million cells were fixed in growth media with 0.4% formaldehyde for 10 min and quenched with 125 mM glycine. Chromatin was released by sequential lysis with cells lysis buffer (10 mM Tris pH 8, 10 mM NaCl, 0.2% IGEPAL CA-630) and nuclear lysis buffer (50 mM Tris pH 8, 10 mM EDTA, 1% SDS). Chromatin was sheared to ~ 300 bp with 12 rounds of sonication (30 s on, 30 s off; Bioruptor, Diagenode) and incubated with 5 μg H3.3 antibody (Abcam, Ab176840) (4 °C, overnight). Samples were immunoprecipitated with Protein A agarose beads (Sigma-Aldrich, 05015979001) and washed sequentially with low salt (20 mM Tris pH 8, 2 mM EDTA, 50 mM NaCl, 1% Triton X-100, 0.1% SDS), high salt (20 mM Tris pH 8, 2 mM EDTA, 500 mM NaCl, 1% Triton X-100, 0.01% SDS), and LiCl buffers (10 mM Tris pH 8, 1 mM EDTA, 0.25 M LiCl, 1% IGEPAL CA-630, 1% sodium deoxycholate). Chromatin was eluted with elution buffer (1% SDS, 100 mM NaHCO_3_), de-crosslinked overnight at 65 °C, incubated with Proteinase K, phenol chloroform extracted and ethanol precipitated.

PML chromatin immunoprecipitation was performed according to a published method [86] with the following modifications. Cells were fixed with 2 mM EGS (Pierce 26,103) in PBS (45 min, RT). Formaldehyde was then added to 1% (20 min, RT) and quenched with 125 mM glycine. Chromatin was sonicated to under 500 bp and lysates were immunoprecipitated with 10 μg PML antibody (Millipore, mab3738). DNA was precipitated with 10 μg of carrier glycogen.

### ChIP-seq library construction

For WT H3.3 R1 and H3.3 K27M H3.3 ChIP R1 samples, libraries were constructed using the Ovation Ultralow System V2 using Nugen protocols (M01379 v3.1) with c-bot clustering using 200 pM of library pool (Illumina Protocol 15,006,165 v02 Jan 2016). Samples were sequenced using 75 bp PE sequencing on an Illumina HiSeq 3000 (Illumina Protocol 15,066,493 Rev A, February 2015).

For all other samples, libraries were constructed Ovation Ultralow System V2 using Nugen protocols (M01379 v3.1). Denaturing and on-board clustering using 2 pM of library pool containing 1% PhiX (Illumina protocol 15,048,776 v15 Apr 2020). Samples were sequenced with 75 SR sequencing using Illumina NextSeq550 High output mode and v2.5 chemistry (Illumina Protocol 15,069,765 v06, Jul 2019).

### ChIP-seq alignment

ChIP-seq of mouse samples were aligned to the mouse genome (mm9) with Bowtie (v1.0.0) [87] with default settings except (m -5) to allow restricted multi-mapping to short repeats. ChIP-seq of human samples were aligned to the human genome (hg38) with Bowtie2 (v 2.4.2) [88] using default parameters. Unmapped and duplicated reads were removed with SAMtools (v 1.10.0) [89] and converted to BigWig files with Wig/BedGraph-to-bigWig converter (Galaxy Version 1.1.1) on usegalaxy.org [90]. Files were visualized in the UCSC browser.

WT H3.3 and PML ChIP-seq and matched input samples were aligned to a repeat database with Repeat Enrichment Estimator v1.0 [91]. In brief, a repeat assembly file was generated using the Repbase database and reads were aligned to this library and counted.

### ChIP-sequencing plots and quantitations

Composite plots of reads across genes were generated with the computeMatrix tool (Galaxy Version 3.3.2.0.0) [92]. WT H3.3 and PML ChIP-seq reads were plotted across all genes over 1 kb in length (*n* = 24,086) in scale-region mode. Genes were fit into 5000 base windows, with additional 5000 bases up and downstream. Two thousand bases were excluded from 5′ and 3′scaling. Plots were generated using plotProfile with default parameters.

For heatmaps, ChIP-seq reads were first normalized for total read counts and converted to bedgraph format with the genomeCoverageBed tool in BEDTools (v 2.20.1) [93], with -scale option set to (1/total read counts). Bedgraph files were converted to bigwig format with Wig/BedGraph-to-bigWig converter (Galaxy Version 1.1.1) on usegalaxy.org [90]. Normalized reads were then plotted around a list of promoters (defined as TSS ± 1 kb) using the computeMatrix tool (Galaxy Version 3.3.2.0.0) [92].

ChIP-seq reads across promoters (TSS ± 1 kb) were calculated using the multiBigwigSummary tool (Galaxy Version 3.3.2.0.0). Active promoters were defined as promoters where H3K4me3 (mouse) or H3K27ac (human) were > 75th percentile.

### Generation of PML depleted human glioma lines

Human H3.3 K27M mutant and H3.3 WT (K27M KO) DIPG XIII glioma cell lines were infected with dCas9-KRAB lentivirus produced in human HEK293 cells, which were transiently transfected with dCas9-KRAB plasmid (Addgene #89,567) and packaging plasmids pMD2.G (Addgene #12,259) and psPAX2 (Addgene #12,260) using Lipofectamine 2000. Cells were subjected to Blasticidin selection (2.5 µg/ml Blasticidin, MP Biomedicals) to generate stable dCas9-KRAB expressing cell clones. Expression of dCas9-KRAB was validated using an anti-Cas9 antibody. sgRNA targeting human PML gene (Additional file 2: Table S3) was designed using the Broad Institute’s GPP sgRNA designer (https://portals.broadinstitute.org/gpp/public/analysis-tools/sgrna-design) and cloned into the FgH1tUTG plasmid (Addgene #70,183) [94] at BsmBI site. dCas9-KRAB repressor expressing H3.3 K27M mutant and H3.3 WT (K27M KO) DIPG XIII cells were infected with sgPML containing FgH1tUTG (or empty vector FgH1tUTG) lentivirus followed by cell sorting to select for GFP-positive cells. PML knockdown was achieved by doxycycline treatment (72 h). Knockdown validation at the protein level was determined by immunofluorescence analysis using an anti-PML antibody.

To direct cell differentiation, H3.3 K27M mutant and H3.3 WT (K27M KO) PML KD DIPG XIII cell lines were seeded in differentiation media with or without 2 µg/ml doxycycline.

### Generation of PML overexpression human glioma lines

Lentiviral particles were produced as previously described except that a CMV-PML isoform 1 (PML-I-pLenti-C-Myc-DDK-IRES-Neo, Genscript) cassette was used in place of dCas9-KRAB. To generate PML I expression construct, PML isoform 1 ORF (Genscript) was cloned into a pLenti-C-Myc-DDK-IRES-Neo expression vector (Origene #PS100081). Lentiviral particles were produced in human HEK293 cells as described above. Briefly, HEK293 cells were transiently transfected with pLenti-C-Myc-DDK-IRES-Neo plasmid and packaging plasmids pMD2.G (Addgene #12,259) and psPAX2 (Addgene #12,260). H3.3 K27M mutant and H3.3 WT (K27M KO) DIPG XIII glioma cell lines were seeded at a density of 3 × 10^5^ cells per well in a 6-well plate and infected with 0.5 ml viral soup and 2 µg/ml polybrene. Transduced cells were selected with G418 (200 µg/ml) after 48 h and G418-resistant PML-I overexpressing cells were used for assays after 10 days in selection media.

### Knockout of IDH1R132H allele in human anaplastic oligodendroglioma line

pSpCas9(BB)-2A-GFP (PX458) was a gift from Feng Zhang (Addgene plasmid # 48,138). Single-guide RNA sequences (Additional file 2: Table S3) were assembled into the pSpCas9(BB)-2A-GFP (PX458) backbone and the construct was nucleofected into BT237 cells according to the manufacturer’s protocol (Lonza). Flow cytometry sorted single GFP + cells in 96-well plates, 72-h post-transfection. Clones were expanded and the target locus sequenced by Illumina MiSeq system and Sanger Sequencing for the target exon to confirm mutation of the IDHR132H mutant allele.

### Arsenic experiments and cell counts

Patient-derived H3.3 K27M mutant and H3.3 WT (K27M KO) DIPG13 and BT245 cell lines were seeded at 2 × 10^4 cells per 12 well. For each line, cells were treated with 1 µM arsenic trioxide (ATO) for 8 days in their respective media. Fresh media was added every 4 days. Duplicate plates were set up for each 4 and 8 day time points. For photography, one plate was coated with laminin (Sigma) and poly-ornithine (Thermo Fisher) for cell attachment. At each time point, cells were photographed with EVOS M5000 and assessed for cell viability by trypan blue staining.

### Direct stochastic optical reconstruction microscopy (dSTORM) experiments

Cells were treated with KaryoMAX Colcemid at a final concentration of 10 µg/ml for 1 h prior to harvest. Cells were then adhered to NuncTM Lab-TekTM II Chambered Coverglass (Thermo Scientific) using Cell-TakTM Cell and Tissue Adhesive (Corning) before fixation and immunolabelling. Fixation was achieved using 3.7% formaldehyde in PBS for 10 min at R.T. followed by washing twice with PBS and then by permeabilization with 0.1% Triton X-100 in PBS for 10 min at R.T. Fixed cells were blocked in 2% bovine serum albumin in PBS for 10 min at R.T., and immunolabelled first with the relevant primary antibody (Millipore mouse anti-PML antibody, 1:1500) for 1 h at 37 °C and then with a secondary antibody Goat anti-mouse conjugated to Alexa Fluor 647 antibody (1:200 dilution; Thermo Scientific) for 45 min at R.T. Labelled cells were washed again and post-fixed with 3.7% formaldehyde in PBS for 5 min at R.T. before storage in 0.05% sodium azide in PBS at 4 °C. Data acquisition and processing: SM imaging was performed on a home-built super-resolution microscope described previously [95, 96], based on an Olympus IX81 inverted fluorescence microscope frame fitted with a TIRF 100X 1.49 NA oil objective, Oxxius 638-nm laser diode and Andor iXon EM-CCD detector. Micromanager [95] was used to set acquisition parameters. Cells were imaged in a switching buffer of 125 mM mercaptoethylamine (MEA) at pH 8.5 (adjusted with 1 M potassium hydroxide) in PBS. Laser power of ~ 3 kW/cm^2^ was used to induce Alexa Fluor 647 SM photoswitching, observed as “blinking” during acquisition. Typically, 10,000 frames of photoswitching SM emissions were acquired at 50 Hz (20 ms exposure). SM point spread functions were identified and fitted by a 2D Gaussian function in rapidSTORM [96] to generate SM coordinate lists. These were then rendered in rapidSTORM to generate final super-resolution images. PML sizes were quantified using ImageJ FIJI software by measuring the diameter of each nuclear body. An artificial threshold was set to eliminate background fluorescence readings and ~ 300 PML bodies were measured for each individual cell line. Data were subsequently collated and plotted using GraphPad Prism Software (V9).

### Supplementary Information

**Additional file 1: Supplementary figures S1-S7.**

**Additional file 2: Tables S1-S3.**

**Additional file 3. **Review history.

## Data Availability

The datasets generated and/or analyzed during the current study have been deposited in the GEO repository under the accession number GSE210190 [97].
