# Supplementary material for: Pediatric glioma histone H3.3 K27M/G34R mutations drive abnormalities in PML nuclear bodies
Source: Genome Biol. 2023 Dec 8;24:284. doi: 10.1186/s13059-023-03122-5 (PMC10704828; doi:10.1186/s13059-023-03122-5)
Supplement: Supplementary file 1 — Additional file 1: Supplementary figures S1-S7. [file 13059_2023_3122_MOESM1_ESM.pdf]

Supplementary Figures

Figure S1. Enrichment of H3.3 and PML ChIP-seq across the genome.

A) Examples of DNA repeats which are not enriched for H3.3 and/or PML in mouse ES cells. GSAT – gamma satellite repeats; IAPEz – intracisternal A-particle; B3 – short interspersed nuclear element (SINE) B3; MusHAL1 – long interspersed nuclear element (LINE); B4A – SINE B4. **B)** Heatmap of H3.3 and PML ChIP-seq, sorted by H3.3 enrichment at randomly selected (*n* = 20,000) 2 kb genome bins. **C)** Scatter plot of H3.3 and PML ChIP-seq reads normalised for total read counts at randomly selected (*n* = 20,000) 2 kb genome bins. **D)** Heatmap of H3.3 and PML ChIP-seq reads at enhancers [96], sorted by H3.3 enrichment. **E)** Scatter plot of H3.3 and PML ChIP-seq reads which mapped to enhancers, normalised for total read counts. **F)** Heatmap of H3.3 and H3K27me3 ChIP-seq at promoters, sorted by H3.3 enrichment. **G)** Scatter plot of H3.3 and H3K27me3 ChIP-seq reads normalised for total read counts at promoters.

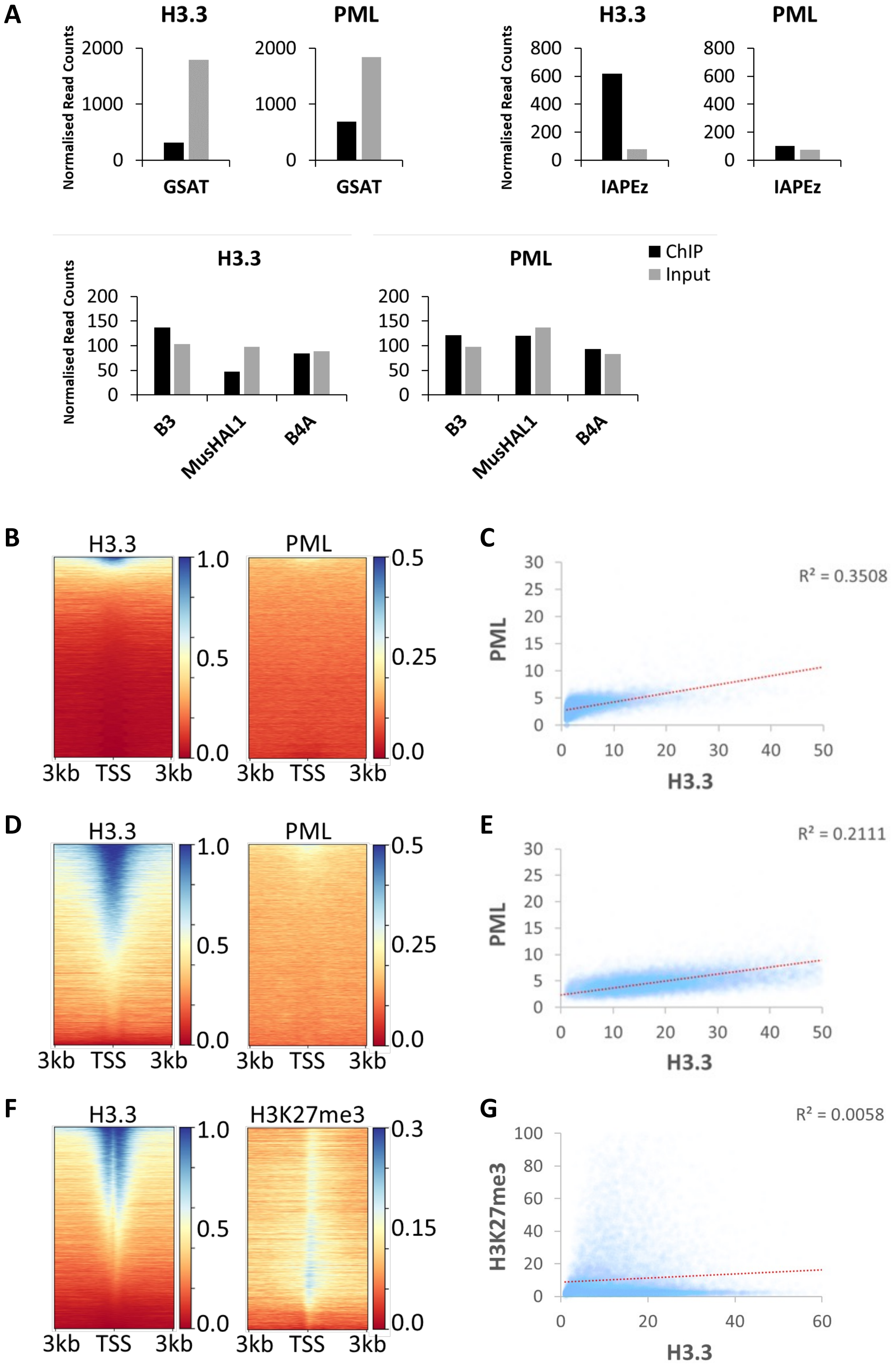
A
H3.3
PML
Normalised Read Counts
2000
1000
0
GSAT
800
600
400
200
IAPEz
ChIP
Input
200
150
100
50
B3
MusHAL1
B4A
B
C
D
E
F
G
1.0
0.5
0.0
0.25
3kb
TSS
R² = 0.3508
R² = 0.2111
R² = 0.0058
30
25
20
15
10
5
40
H3K27me3
0.3
0.15
100
80
60

Fig S1

Figure S2. Creation and characterisation of mouse ES cells with a single copy H3.3 K27M mutation. A) Targeting strategy for the expression of an H3.3 K27M mutant cds driven by the endogenous *H3f3a* promoter. The promoter-less H3.3K27 and -M27 minigenes/cassettes were each comprised of the cDNA, *H3f3a* UTR and the late-SV40 poly(A) signal sequence. For positive selection, the neo cds was driven by the mouse Pgk1 promoter and terminated with the bovine growth hormone poly(A) signal sequence. For negative selection, the diptheria toxin A-chain cds was regulated by these same elements. The three cassettes were substituted for exon2 (e2) to bring the H3.3K27 minigene under endogenous regulatory control (c allele). On exposure to Cre (+Cre) the H3.3K27 and neo cassettes were excised, bringing the H3.3M27 cassette under regulatory control (- allele). Transcription (green arrow), no transcription (red cross). Location of Southern blot probes and expected band sizes are shown. **B)** Southern blots of clones which are homozygous for WT *H3f3a*; heterozygous for the conditional allele; and heterozygous for the H3.3 K27M mutant allele. Southern blots of subclones resistant (res.) and sensitive (sen.) to G418. Predicted band sizes were seen, demonstrating conservative recombination for clones. **C)** Immunostaining and dSTORM analysis of PML (orange) in WT, H3.3 K27M, H3.3 G34R, and H3.3 G34R/ATRX KO (double mutant; DM). Colour gradient represents the relative intensities of PML-NBs. Scale bar 1 µM. **D)** Western blots analysis of WT, H3.3 K27M (clone #1 and #2), H3.3 G34R, and H3.3/G34R (double mutant; DM) mouse ES cell lines using antibodies against PML and actin.

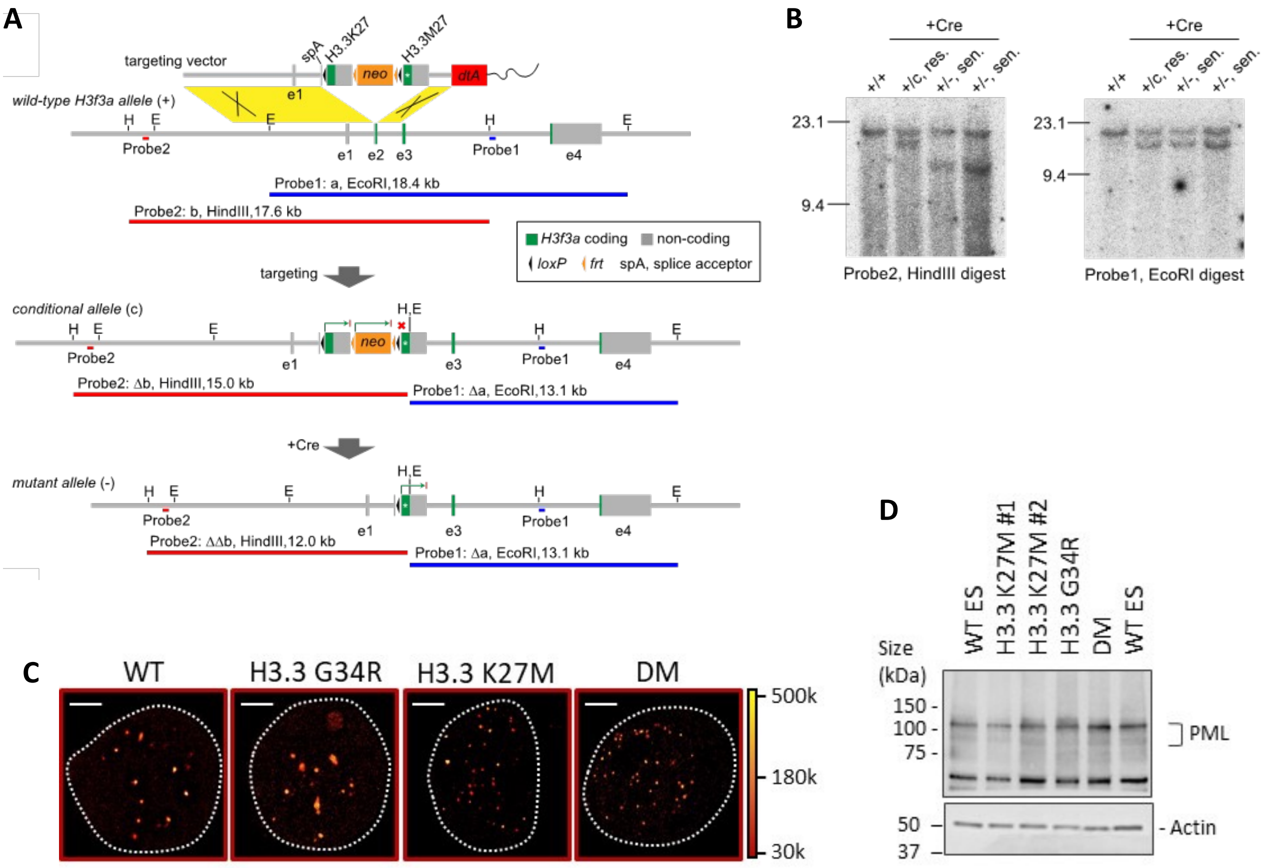
A
targeting vector
spA
H3.3K27
H3.3M27
neo
dtA
wild-type H3f3a allele (+)
H
E
e1
e2
e3
e4
Probe2
Probe1
Probe1: a, EcoRI,18.4 kb
Probe2: b, HindIII,17.6 kb
H3f3a coding
non-coding
loxP
frt
spA, splice acceptor
targeting
conditional allele (c)
H,E
Probe2: Δb, HindIII,15.0 kb
Probe1: Δa, EcoRI,13.1 kb
+Cre
mutant allele (-)
Probe2: ΔΔb, HindIII,12.0 kb
Probe1: Δa, EcoRI,13.1 kb
B
+Cre
+/+
+/c, res.
+/-, sen.
+/-, sen.
23.1
9.4
Probe2, HindIII digest
Probe1, EcoRI digest
C
WT
H3.3 G34R
H3.3 K27M
DM
500k
180k
30k
D
Size (kDa)
WT ES
H3.3 K27M #1
H3.3 K27M #2
H3.3 G34R
DM
WT ES
150
100
75
50
37
PML
Actin

Fig S2

Figure S3. Western blots of DIPG XIII and BT245 H3.3 K27M mutant and H3.3 WT (K27M KO) lines with antibodies against H3K27M and actin.

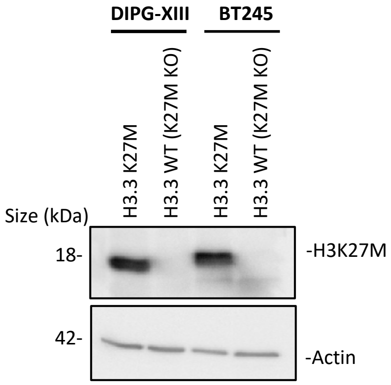

Figure S4. Western blot and immunofluorescence in H3.3 K27M mutant patient-derived glioma cells. A) Western blots of H3.1 K27M [SU_DIPG_36 (#1), ICR_B184_2D (#2), SU_DIPG_33 (#3), SU_DIPG_4 (#4)] human patient derived glioma cell lines with antibodies against H3K27M and H3. **B)** Immunofluorescence of PML (red) and ATRX (green) and in H3.3 K27M (left panels) and H3.1 K27M (right panels) human patient derived glioma cell lines. Scale bar 2 µM.

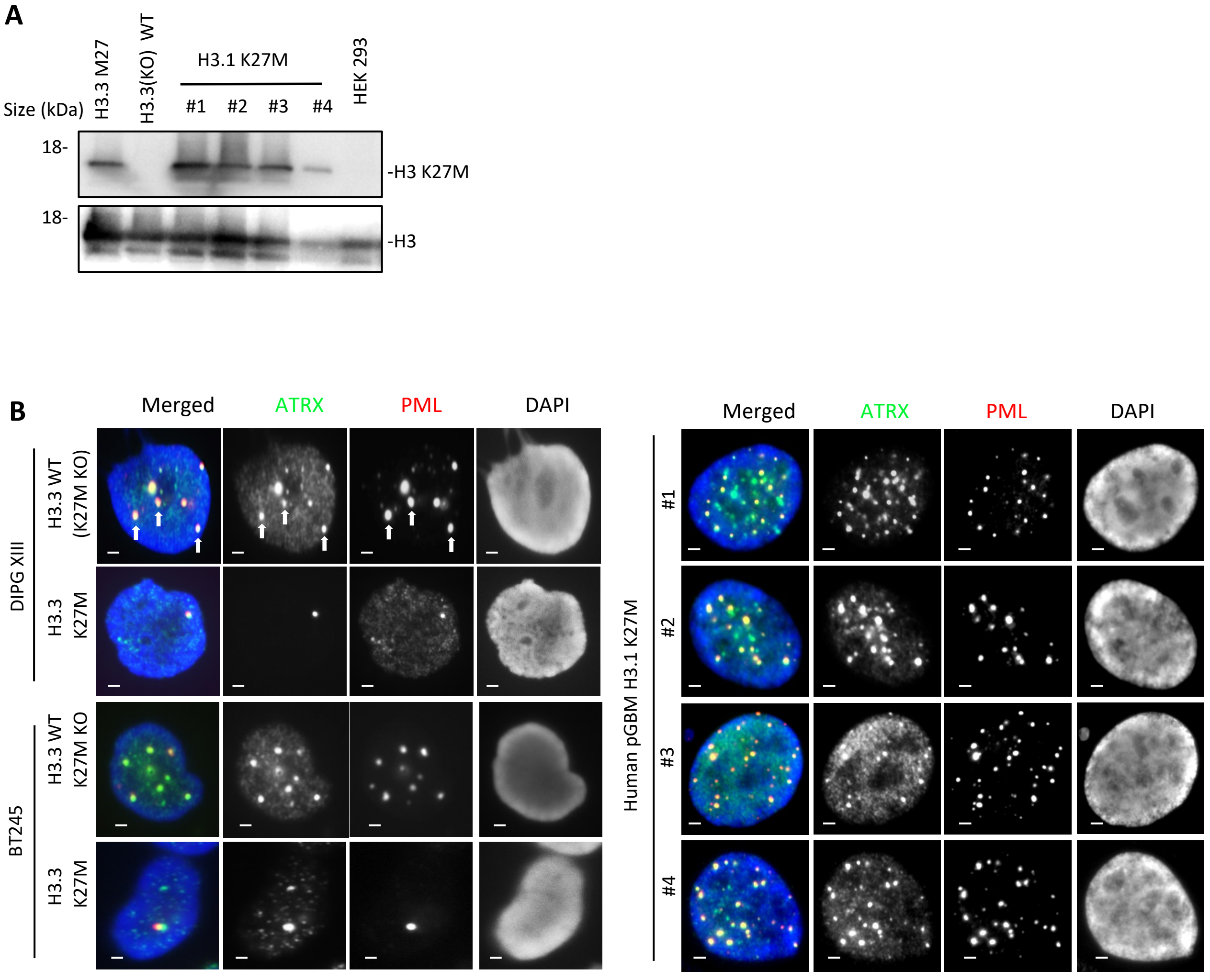

Fig S4

Figure S5. PML knockdown inhibits differentiation in H3.3 K27M KO glioma cells. A) Western blots of dCAS9 Krab repressor in H3.3 K27M mutant and H3.3 WT (K27M KO) DIPG XIII human patient derived glioma cell lines. **B)** Western blot of PML and actin in two H3.3 K27M and H3.3 WT (K27M KO) clones (clones #1 and #2) ± doxycycline induced knockdown of PML. **C)** Immunofluorescence staining of GFAP glial marker (green) and β-tubulin (red) of a second H3.3 WT (K27M KO) and mutant H3.3 K27M clone #2 with and without PML knockdown, over 7 and 14 days of culture in differentiation media.

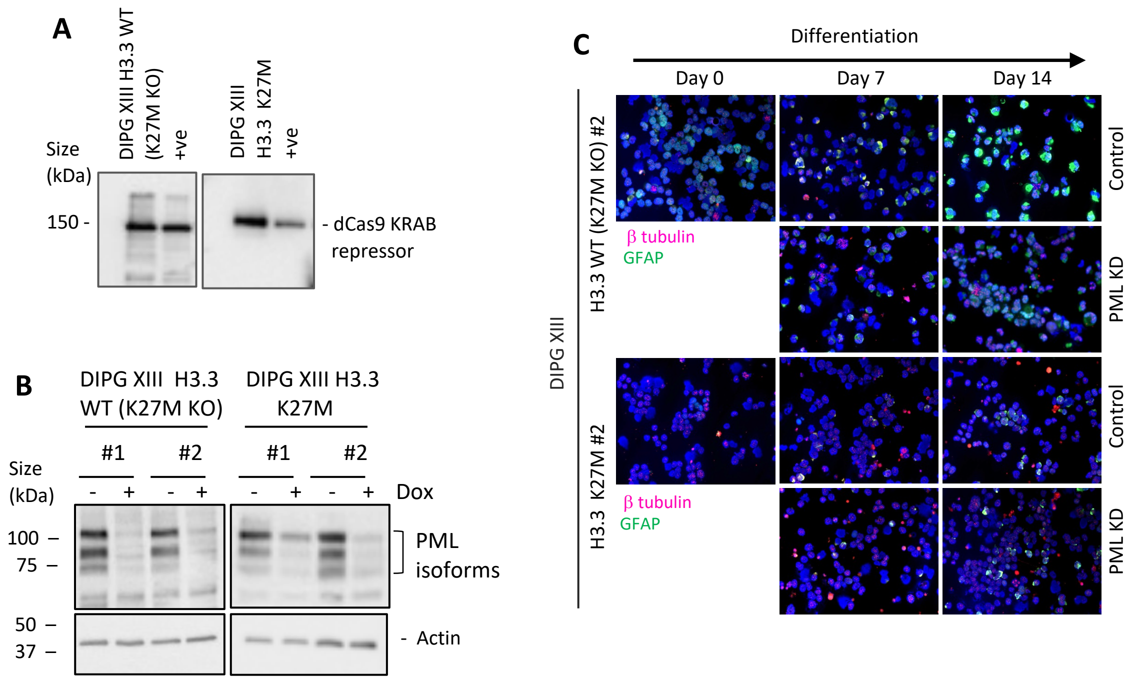

Fig S5

Figure S6. Overexpression of PML reduces sensitivity to arsenic trioxide and partially restores differentiation capacity in H3.3 K27M KO glioma cells. Western blots of PML in **A)** WT H3.3 (K27MKO) and **B)** WT H3.3 (K27MKO) and H3.3 K27M DIPG XIII patient-derived glioma cells were treated with 1 μM ATO for 1, 4, and 24 hours. Cell lysates were prepared from the respective samples using the CSK buffers. Western Blot analysis of PML protein extracted from the various soluble (S1 – S3) and insoluble (Pellet; P) fractions. Histone H3 staining was included as a loading control. **C-D)** Immunostaining of PML (green) and centromere (crest; red) in WT H3.3 (K27MKO) (C) and H3.3 K27M (D) DIPG XIII patient-derived glioma cell lines treated with 1 μM ATO for 1, 4, and 24 hours. **E**) Immunostaining of H3.3 K27M (green) and PML (red) in H3.3 K27M paediatric glioma cell lines SU-DIPG 17, JHH-DIPG-1 and P002306. **F)** Cell counts of H3.3 K27M paediatric glioma cell lines SU-DIPG 17, JHH-DIPG-1 and P002306 treated with 1 μM of ATO for 0, 4, and 8 days. **G-H)** Cell counts of DIPG XIII H3.3 WT (K27M KO) (G) and H3.3 K27M (H) paediatric glioma cells with and without exogenous PML overexpression (+PML) treated with 1 μM of arsenic trioxide for 0, 4, and 8 days. Untreated cells are shown as controls. Points and error bars represent the mean average and standard deviation of three independent experiments. *P*-values were calculated using a two-tailed Student's *t*-test (ns = not significant, *$P < 0.005$, **$P < 0.001$, ***$P < 0.0005$).

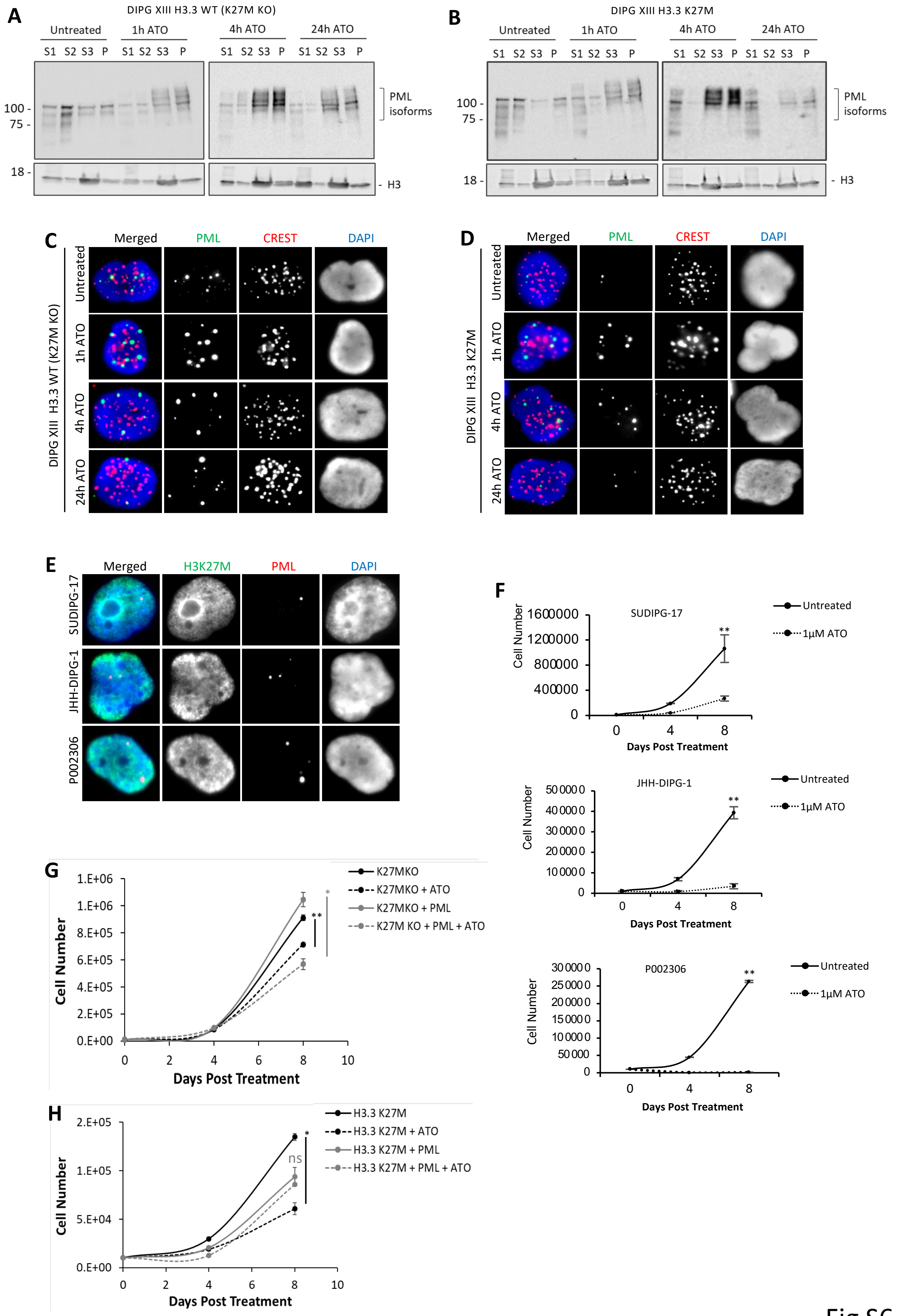
A
DIPG XIII H3.3 WT (K27M KO)
Untreated
1h ATO
4h ATO
24h ATO
S1 S2 S3 P
PML isoforms
100 -
75 -
18 -
- H3
B
DIPG XIII H3.3 K27M
C
Merged
PML
CREST
DAPI
DIPG XIII H3.3 WT (K27M KO)
D
DIPG XIII H3.3 K27M
E
H3K27M
SUDIPG-17
JHH-DIPG-1
P002306
F
Cell Number
Days Post Treatment
Untreated
1μM ATO
G
K27MKO
K27MKO + ATO
K27MKO + PML
K27M KO + PML + ATO
H
H3.3 K27M
H3.3 K27M + ATO
H3.3 K27M + PML
H3.3 K27M + PML + ATO
ns

Fig S6

Figure S7. Characterisation of IDH1 R132H mutant mouse ES cells and human anaplastic oligodendroglioma cell lines. A) Immunostaining and dSTORM analysis of PML (orange) in WT and IDH R132H mouse ES cells. Colour gradient represents the relative intensities of PML-NBs. Scale bar 1 µM. **B)** Western blot of PML in WT and IDH1 R132H mutated mouse ES cells. **C)** DNA sequencing chromatogram of BT237 human anaplastic oligodendroglioma cell lines. Human BT237 IDH1 R132H oligodendroglioma cell line and the isogenic R132H KO line were subjected to Sanger sequencing. The sequencing chromatograms showed the presence of the mutated Histidine codon (CAT, highlighted in blue) in both BT237 IDH1 R132H and IDH1 WT (R132H KO) cell lines, and a two-base deletion (AT deletion) in IDH1 WT (R132H KO) cells. The AT deletion caused a frameshift in the R132H allele, thereby removal of the IDH1 R132H allele in IDH1 WT (R132H KO) line. **D)** Western blots of BT237 IDH1 R132H and IDH1 WT (R132H KO) cell lines using antibodies against PML and actin.

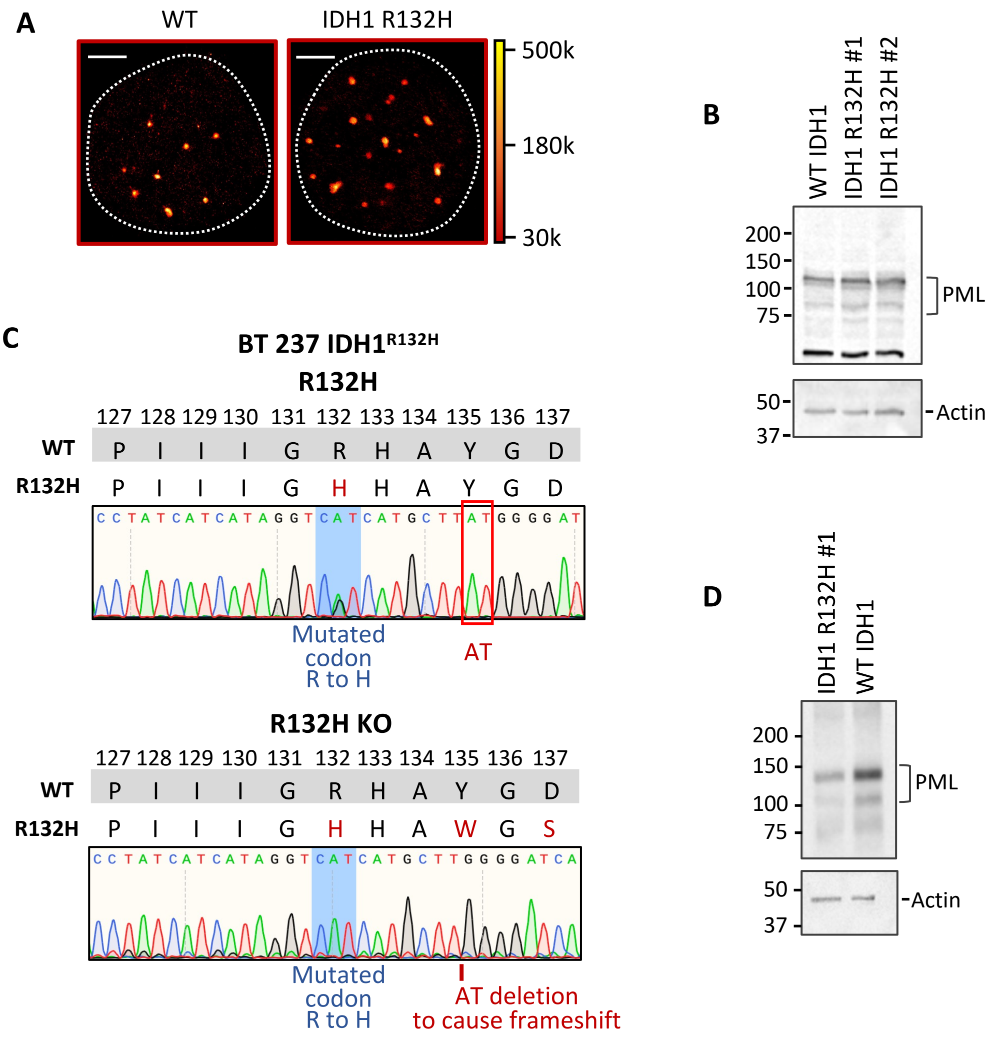

Fig S7
