# Supplementary material for: Pediatric glioma histone H3.3 K27M/G34R mutations drive abnormalities in PML nuclear bodies
Source: Genome Biol. 2023 Dec 8;24:284. doi: 10.1186/s13059-023-03122-5 (PMC10704828; doi:10.1186/s13059-023-03122-5)
Supplement: Supplementary file 2 — Additional file 2: Tables S1-S3. [file 13059_2023_3122_MOESM2_ESM.pdf]

Table S1

Primer sequences for PCR amplification and RT-qPCR

	Primer sequences for PCR amplification and RT-qPCR analysis	
GFAP	*5′* GCAGAAGCTCCAGGATGAAA *3′*	*5′* GCTTCATCTGCTTCCTGTCTAT *3'*
GAPDH	5' CGAGATCCCTCCAAAATCAA 3'	5' TTCACACCCATGACGAACAT 3'

Table S2

List of antibodies used

Antibodies	Source	Antibodies	Source
H3	Abcam, ab1791	ATRX	Santa Cruz Biotechnology, sc15408
H3.3	Abcam, ab176840	DAXX	Santa Cruz Biotechnology, sc-7152
H3K27M	Abcam, ab190631	PML (for use in mouse cells)	Millipore, MAB3738
β Actin (AC15)	Santa Cruz Biotechnology, sc6987	PML (for use in human cells)	Abcam, ab179466 (ChIP, WB)<br>Abcam, ab96051 (IF)
dCas9 KRAB repressor	Abcam, ab191468	Myc (clone 9E10)	Sigma, M4439
GFAP	Cell Signalling Technology, 12389	Flag tag (M2)	Sigma, F3165
Goat anti Rabbit IgG, HRP conjugate	Merck Millipore, AP187P	Donkey anti-Mouse IgG (H+L), Alexa Fluor 488	Thermo Fisher Scientific, A-21202
Donkey anti-Mouse IgG, HRP conjugate	Merck Millipore, AP192P	Donkey anti-Rabbit IgG (H+L), Alexa Fluor 488	Thermo Fisher Scientific, A-21206
Goat anti-mouse IgG (H+L) Alexa Fluor 647	Thermo Fisher Scientific, A-32728	Chicken anti-Rabbit IgG (H+L), Alexa Fluor 594	Thermo Fisher Scientific, A-21442
Donkey anti-Mouse IgG (H+L), Alexa Fluor 594	Thermo Fisher Scientific, A-21203		

Table S3

Primer sequences for sgRNA

	Primer sequences for generating the constructs
PML Knockdown sgRNA	*5′* tcccGTGAAGAGAAGCTGAAGGGG *3′*<br>*5′* aaacCCCCTTCAGCTTCTCTTCAC *3′*
IDH1R132H knockout sgRNA	*5'* caccgCATAGGTCATCATGCTTATG *3'*<br>*5'* aaacCATAAGCATGATGACCTATGc *3'*
