# Supplementary material for: Pediatric glioma histone H3.3 K27M/G34R mutations drive abnormalities in PML nuclear bodies
Source: Genome Biol. 2023 Dec 8;24:284. doi: 10.1186/s13059-023-03122-5 (PMC10704828; doi:10.1186/s13059-023-03122-5)
Supplement: Supplementary file 3 — Additional file 3. Review history. [file 13059_2023_3122_MOESM3_ESM.docx]

Review History

**First round of review**

**Reviewer 1**

**Were you able to assess all statistics in the manuscript, including the appropriateness of statistical tests used?**

Yes, and I have assessed the statistics in my report.

**Comments to author:**

In the manuscript entitled: "Paediatric Glioma Histone H3.3 K27M/G34R Mutations Drive Abnormalities in Phase-Separated PML NuclearBodies", Voon et al set to understand the link between oncohistone mutations and PML-NBs biology. The authors investigate H3.3 and PML colocalization focusing specifically on telomeres and subsets of promoters. They introduce mutations into endogenous H3.3 gene to produce onco-histones in mESCs. In these cells, they find that mutations disrupt the number and size of PML bodies, an observation that they later reproduce in defined tumor-derived cell lines harboring typical oncohistone mutation, as well as in IDH1-mutated cells. Finally, the authors show that failure to differentiate in oncohistone tumor-derived cells is a phenotypic alteration shared with cells depleted for PML, and that all these cells ultimately proved sensitive to arsenic trioxide treatment.

So called oncohistone mutations gained a lot of attention in the last decade and it is still not clear how they drive such cell type-specific tumorigenesis. The link between PML-NBs and the cancerous properties of the oncohistone explored in this manuscript is therefore certainly of interest for the wider scientific community. However, while the approach is certainly interesting, a series of controls and analysis are missing for the manuscript to be shared with the community. There is also a need to add coherence since certain experiments are performed under some conditions, but not others. Moreover, the manuscript largely describes dynamics of PML-NBs in certain cell types, yet a functional analysis or attempts to make a stronger connection between these changes and tumorigenesis is still missing. As it stands the manuscript, with the necessary control and analysis could however represent an interesting piece to format as a short communication, which is one of the formats available in Genome Biology.

- Firstly, authors always refer to oncohistones "disrupting PML-NBs" - while this is technically true, in different cell types and different mutations, authors observe opposite effects (decrease vs increase in numbers) on PML-NBs. Moreover, these changes in some cases correlate positively, and in some negatively with H3.3 changes (on promoters where H3.3 and PML co-reside). It will be important for the authors to be more precise and at least offer an interpretation for why these diverse changes could be a manifestation of the same mechanism.

- Second, the genomic data need more careful analysis. It is not clear whether the data were normalized to the input, which is critical for the interpretation. When comparing different cell types certain data sets are missing (for example PML in oncohistone tumor-derived lines) and that makes it difficult to analyze. And finally, even though the authors identify subset of promoters with co-occupancy of PML and H3.3, they do not explore it in any way to understand the significance of this finding - what are these promoters? Does the transcriptional output linked to these regions change when oncohistone is introduced?

Finally, should they wish to aim for a longer manuscript, then several major considerations should be fully taken into account and addressed. Here the final major concern is related to linking PML changes to tumorigenesis in case of oncohistone: While the experiment performed in Figure 5 shows that both PML KD and oncohistone lead do failure in differentiation, there is no causal link between the two. A relatively simple PML overexpression experiment could bring such causative data and bring more strength to author's arguments.

With the necessary additions and corrections, the manuscript could form a very exciting contribution. Hopefully, the authors will be able to use the detailed critical comments below to improve their manuscript.

To make it easier to follow, the necessary experimental work for revisions are indicated below linked to the data provided figure by figure:

- Figure 1 - it is important to clarify the normalization of the ChIP experiment - is the data in heatmaps normalized to the input? In the current state the Materials and Methods section is not clear about it, and such normalization is critical to make conclusions about signal level changes within samples;

- Figure 2 - there are interesting inconsistencies between the cell lines that perhaps should be explored, rather than pulled together in "PML-NBs disruption phenotype"

* (Panel C) the three cell lines seems to have distinct changes in the number of PML-NBs - K27M leads to increased number, G34R leads to decreased number, and DM cell show what looks like two populations of cells - some with numbers similar to WT, and some with much higher PML-NBs number - do the authors have any hypothesis on why such distinct presentation could be leading to the same defects in the cell physiology? Especially that in APL the number of PML-NBs consistently increases, so to draw parallels between the two pathologies it would be important to offer interpretation of such discrepancy

* (Panels E-G) there is a very interesting change in pattern of the H3.3 peaks between WT and oncohistone cells - in WT there are two clear peaks, but in mutants the signal becomes one, central peak, as if upon mutation the nucleosome positioning changed in this region - is this a phenomenon specific to the promoters? Also, it would be interesting to see how is the H3.3 and PML signal affected on telomeres to understand if the level effects observed on heat maps are global, or specific to these regions.

* (Panels H-J) In all the mutants the H3.3 signal at promoters increases, but the PML signal strength appears to be independent from this - it decreases in K27M, stays the same in G34R and increases in DM; wouldn't then the interpretation be that changes in H3.3 and PML occupancy in these cells are independent from each other?

Authors' conclusion from the figure is that "H3.3 point mutations severely disrupt the formation and localization of PML-NBs" - it is correct, but it does not accurately reflect the complexity of results presented, and this should be amended.

- Figure 3 - In the case of tumor-derived cell lines, the authors observe opposite effect when compared to mESCs results when it comes to number of PML-NBs - they increased in mESCs and decreased in the tumor-derived lines. In both cases however the levels of H3.3 increases over the promoter - it would be important to know how do the PML levels change in these regions? Doesn't the fact that changes in H3.3 and PML-NBs are not well correlated with each other argue that these two factors are not strictly related?

- Figure 4 - here the quantification of the phenotype "number of PML bodies" will be necessary - based on the representative images provided it seems like there is a rather strong phenotype of increased number of PML-NBs in H3.1 K27M cell lines as compared to WT H3.3 lines - is this really the case? A great deal of effort was placed in previous panels to document the PML-NBs changes in H3.3 mutants and as is, it is not convincing to rule out any differences in H3.1 mutant based on unquantified images only

- Figure 5 - the fact that depletion of PML leads to failure in differentiation is a strong and suggestive result; however, it does not directly link oncohistone tumor-derived cells phenotype to disruption of PML bodies; taking into account that in tumor-derived cells the number and size of PML-NBs indeed decrease, maybe rescuing the non-differentiation phenotype in these cells via PML overexpression would be a better suited experiment, as it could provide direct evidence that oncohistone-driven phenotype are indeed linked to its effect on PML-bodies

- Figure 6 - this is an interesting experiment in light of Figure 5 - if the authors are correct, that the therapeutic effect of ATO in the case of oncohistone tumor-derived cells is due to degradation of PML, then the PML KD experiment should give rise to similar results in a cell viability assay - is that the case? That would be an additional and strong evidence that cell fate aberrations in oncohistone tumor-derived cell lines are linked with PML function.

**Reviewer 2**

**Were you able to assess all statistics in the manuscript, including the appropriateness of statistical tests used?**

Yes, and I have assessed the statistics in my report.

**Comments to author:**

The manuscript by Voon et al. discusses the point mutations in H3.3 disrupt the formation of PML nuclear bodies and prevents differentiation down glial lineages. The tumor cells carrying H3.3 mutations are sensitive to drugs that target PML bodies. The point mutations in IDH1/2 are also associated with disrupting the formation of PML bodies. The manuscript is well written and attractive. Here are some comments:

1. The authors demonstrated that H3.3 K27M patient-derived glioma cells are sensitive to ATO through in vitro experiment. Then, how about the results by in vivo assay?

2. As mentioned by the authors that IDH1 mutation could have an influence on PML-NBs, and IDH1 mutated cells were likely to be sensitive to treatment with ATO. So, it would be interesting to compare the response of the cell lines with H3.3 mutation or IDH1 mutation to ATO treatment.

3. The Figures are unclear especially in figure 6 and figure 7.

**Authors Response**

**Point-by-point responses to the reviewers’ comments:**

**Reviewer #1 General comments:**

In the manuscript entitled: "Paediatric Glioma Histone H3.3 K27M/G34R Mutations Drive Abnormalities in Phase-Separated PML Nuclear Bodies", Voon et al set to understand the link between oncohistone mutations and PML-NBs biology. The authors investigate H3.3 and PML colocalization focusing specifically on telomeres and subsets of promoters. They introduce mutations into endogenous H3.3 gene to produce onco-histones in mESCs. In these cells, they find that mutations disrupt the number and size of PML bodies, an observation that they later reproduce in defined tumor-derived cell lines harboring typical oncohistone mutation, as well as in IDH1-mutated cells. Finally, the authors show that failure to differentiate in oncohistone tumor-derived cells is a phenotypic alteration shared with cells depleted for PML, and that all these cells ultimately proved sensitive to arsenic trioxide treatment.

So called oncohistone mutations gained a lot of attention in the last decade and it is still not clear how they drive such cell type-specific tumorigenesis. The link between PML-NBs and the cancerous properties of the oncohistone explored in this manuscript is therefore certainly of interest for the wider scientific community. However, while the approach is certainly interesting, a series of controls and analysis are missing for the manuscript to be shared with the community. There is also a need to add coherence since certain experiments are performed under some conditions, but not others. Moreover, the manuscript largely describes dynamics of PML-NBs in certain cell types, yet a functional analysis or attempts to make a stronger connection between these changes and tumorigenesis is still missing. As it stands the manuscript, with the necessary control and analysis could however represent an interesting piece to format as a short communication, which is one of the formats available in Genome Biology.

Firstly, authors always refer to oncohistones "disrupting PML-NBs" - while this is technically true, in different cell types and different mutations, authors observe opposite effects (decrease vs increase in numbers) on PML-NBs. Moreover, these changes in some cases correlate positively, and in some negatively with H3.3 changes (on promoters where H3.3 and PML co-reside). It will be important for the authors to be more precise and at least offer an interpretation for why these diverse changes could be a manifestation of the same mechanism.

Second, the genomic data need more careful analysis. It is not clear whether the data were normalized to the input, which is critical for the interpretation. When comparing different cell types certain data sets are missing (for example PML in oncohistone tumor-derived lines) and that makes it difficult to analyze. And finally, even though the authors identify subset of promoters with co-occupancy of PML and H3.3, they do not explore it in any way to understand the significance of this finding - what are these promoters? Does the transcriptional output linked to these regions change when oncohistone is introduced?

Finally, should they wish to aim for a longer manuscript, then several major considerations should be fully taken into account and addressed.  Here the final major concern is related to linking PML changes to tumorigenesis in case of oncohistone: While the experiment performed in Figure 5 shows that both PML KD and oncohistone lead do failure in differentiation, there is no causal link between the two. A relatively simple PML overexpression experiment could bring such causative data and bring more strength to author's arguments.

With the necessary additions and corrections, the manuscript could form a very exciting contribution. Hopefully, the authors will be able to use the detailed critical comments below to improve their manuscript. To make it easier to follow, the necessary experimental work for revisions are indicated below linked to the data provided figure by figure:

*Response: We are grateful for these comments. In response to these comments, we have provided a more comprehensive explanation of our observations and have broadened our discussion regarding the intricate interactions between H3.3 mutations and the functionality of PML nuclear bodies (PML-NBs). Furthermore, we have incorporated new data in our revised study: (i) additional H3.3 K27M patient-derived cell lines showing sensitivity to arsenic trioxide (ATO) (****Fig S6 C-D****), (ii) overexpression of PML partially restores the differentiation phenotype and reduces arsenic sensitivity in H3.3 K27M DIPG XIII glioma cells (****Fig 5C-D, S6E-F****). These new results underscore our hypothesis that H3.3 mutations exert their effects by disrupting the normal functioning of PML bodies.*

*Since all are raised again in more detail below, we provide point-by-point replies in the subsequent text. In addition, we have highlighted in text (in red) the changes made in the manuscript. Please note however that given the extent of our experiments (model systems and approaches) and data, we do prefer maintaining a full-length manuscript rather than a short communication.*

**Specific comments**

1) Figure 1 - it is important to clarify the normalization of the ChIP experiment - is the data in heatmaps normalized to the input? In the current state the Materials and Methods section is not clear about it, and such normalization is critical to make conclusions about signal level changes within samples;

*Response: We apologise for not making it clear the normalization of the ChIP experiment. We have now clarified this in the methods (page 24). In brief, for heatmaps, ChIP-seq reads were first normalised for total read counts and converted to bedgraph format with the genomeCoverageBed tool in BEDTools (v 2.20.1), with -scale option set to (1/total read counts). Bedgraph files were converted to bigwig format* *with Wig/BedGraph-to-bigWig converter (Galaxy Version 1.1.1) on usegalaxy.org. Normalised reads were then plotted around a list of promoters (defined as TSS ± 1 kb) using the computeMatrix tool (Galaxy Version 3.3.2.0.0).*

2) Figure 2 - there are interesting inconsistencies between the cell lines that perhaps should be explored, rather than pulled together in "PML-NBs disruption phenotype".

*Response: We agree that there are a number of interesting observations that require further exploration. However, the interplay between ATRX, H3.3, DAXX and PML is highly complex and dynamic, without even accounting for the many other proteins known to localise to PML bodies. The experiments required to clearly delineate these relationships are beyond the scope of this study. However, we agree that a discussion of our results could be improved, which we have now done, in conjunction with discussion points for point #3 (see below).*

3) Figure 2 Panel C - The three cell lines seems to have distinct changes in the number of PML-NBs - K27M leads to increased number, G34R leads to decreased number, and DM cell show what looks like two populations of cells - some with numbers similar to WT, and some with much higher PML-NBs number - do the authors have any hypothesis on why such distinct presentation could be leading to the same defects in the cell physiology? Especially that in APL the number of PML-NBs consistently increases, so to draw parallels between the two pathologies it would be important to offer interpretation of such discrepancy.

*Response: The normal biological functions of PML-NBs are still not well understood and there are currently too many unknown variables to speculate on how differences in PML-NBs translate to cellular phenotypes. PML bodies are complex and dynamic entities that host a wide range of unrelated proteins****^1,2^.*** *Additionally, several factors have made it very challenging to fully characterise PML-NBs, including: i) Multiple PML isoforms with distinct functions that are not fully understood; ii) A diverse array of PML-interacting proteins that vary according to cell type and stress conditions, many which interact only transiently with PML; iii) The transient and dynamic nature of PML-NBs themselves, which reversibly polymerise and dissociate in response to stimuli such as cell cycle stage or environmental stress (e.g. oxidation, viral infection); iv) The insoluble nature of polymerised PML shells (refer to our experiments in* ***Fig. S6A-B****). Notwithstanding, while it remains risky to speculate on how dysfunctional PML-NBs may lead to differentiation failure with any degree of accuracy, our data clearly show that functional PML-NBs are required for differentiation.*

*Characterizing PML-NBs based solely on staining patterns can be challenging. The diffuse staining pattern of PML in APL can be seen from two perspectives: one might interpret it as an indication of a greater number of PML-NBs, but equally, given their unusually small size, it's plausible to perceive these cells as lacking PML-NBs. Notably, the latter interpretation gains support from the observation that PML-NBs can re-form when PML-mutated cells are treated with ATO. The current prevailing hypothesis is that PML-RARα mutations hinder the formation and maturation of functional PML-NBs****^3,4^****. ATO is thought to play a biphasic role in overcoming this hurdle by first enhancing the formation of functional PML-NBs, then promoting the degradation of mature PML-NBs****^3,4^.***

*As PML-NBs undergo a cycle of aggregation, maturation and degradation, it is possible that the PML-RARα mutations and the different H3.3 mutations interfere with different points of this cycle. Speculatively, the specific point of interference (aggregation/maturation/degradation) could give rise to* *larger/smaller, more/less PML-NBs even though the final outcome is the same – an arrest in PML-NB cycling that leads to failure to differentiate.*

*We have addressed this in the discussion section in conjunction with point 2 (see below).*

*Text added in the discussion (page 16):*

*We found that the two H3.3 point mutations (G34R/K27M) which are common in paediatric brain cancers disrupted the formation of PML-NBs. Interestingly, although both H3.3 mutations alter the size and number of PML bodies relative to WT H3.3, the specific effects vary depending on the point mutation (G34R, K27M), the presence of additional mutations (e.g. ATRX), and the cell type (human glioma, mouse ES cells). As PML-NBs undergo a cyclical process of aggregation, maturation and degradation^5-7^, the variation in size and numbers of PML-NBs between mutants could be due to interference with different stages of this process. For example, the PML-RARα mutation appears to block the formation of normal PML-NBs as treatment with ATO first promotes the formation of functional PML-NBs and subsequent degradation of hypersumoylated PML-NBs^8-10^. The H3.3/ATRX mutations could interfere with different points of the nucleation/maturation/degradation cycle. ATO appears to play a general role in promoting nucleation and degradation of PML-NBs and can therefore override these hurdles regardless of the phase or specific cause of PML-NB cycle arrest.*

*Although we are at present not able to account for the exact mechanisms behind PML disruptions, it is nonetheless clear that (i) normal functional PML-NBs are required for differentiation, and (ii) H3.3 mutations interfere with this process. Considering the highly complex and dynamic interplays between H3.3, DAXX, ATRX and PML-NBs, several reasons may account for this, but the most likely explanation is that the different H3.3 mutations may affect the binding of H3.3 interacting proteins. PML-NBs are dynamic structures that host a diverse array of constantly changing nuclear proteins, so that PML-NBs may have different compositions within a cell****^1,2,11,12^.*** *The mutated residues on H3.3 could potentially interfere with a range of PML-associated proteins which promote or disrupt the nucleation or degradation of PML-NBs. If the mutated histones are acting through protein partners which reside in PML bodies, this may explain why it has been unexpectedly difficult to identify binding partners that are directly affected by H3.3 mutations in vivo. The insoluble nature of PML-NBs is an additional hurdle that can be overlooked with conventional biochemistry protocols. It will be very interesting to see if potential mutant H3.3 interacting partners can be identified using techniques which are optimised for extraction of PML-NBs.*

4) Figure 2 Panels E-G - There is a very interesting change in pattern of the H3.3 peaks between WT and oncohistone cells - in WT there are two clear peaks, but in mutants the signal becomes one, central peak, as if upon mutation the nucleosome positioning changed in this region - is this a phenomenon specific to the promoters?

*Response: The nucleosome positioning profile is specific to promoters. As the reviewer states, this may be due to a change in nucleosome positioning at promoters. However, there are also a number of other possible explanations – for example, there may be a very high turnover of H3.3 at these sites and rapid exchange with Pol II. We agree this point is interesting, and we have added these elements to the results section and speculate on possible causes.*

*Text added to the results section (page 8):*

*In addition, the H3.3 mutations also appear to disrupt the H3.3 distribution profile at transcriptional start sites (TSS). ChIP-seq of H3.3 in WT cells shows nucleosome depleted regions at TSS which is lost in the H3.3 mutants. This may be due to very high levels of H3.3 at promoters or rapid exchange of H3.3 nucleosomes and Pol II at these sites.*

5) Figure 2 - Also, it would be interesting to see how is the H3.3 and PML signal affected on telomeres to understand if the level effects observed on heat maps are global, or specific to these regions.

*Response: Based on the CHIP-seq analysis, the telomeres appear to behave quite differently from the promoters (shown below). PML enrichment at telomeres remains largely unaltered in all three mutant lines while H3.3 is lost at K27M and G34R/ATRX (DM) mutants, and increased in G34R mutants. One possible reason for this discrepancy is that ATRX is required for H3.3 deposition specifically at the telomeres. This is certainly the case for the G34R/ATRX KO double mutant (DM), where there is a sharp decrease in H3.3 relative to both WT and G34R cells. Interestingly, the K27M mutant appears to exclude ATRX from PML-NBs (****Fig. 2B****) for reasons that are currently unclear, which may account for the loss of H3.3 from telomeres in these mutants.*

*As this is not a main focus of the paper, and we are not able to fully account for these observations at this point, we would prefer to not include this data in the paper at this point as it may detract from the clarity of the paper.*

6) Figure 2 Panels H-J - In all the mutants the H3.3 signal at promoters increases, but the PML signal strength appears to be independent from this - it decreases in K27M, stays the same in G34R and increases in DM; wouldn't then the interpretation be that changes in H3.3 and PML occupancy in these cells are independent from each other? Authors' conclusion from the figure is that "H3.3 point mutations severely disrupt the formation and localization of PML-NBs" - it is correct, but it does not accurately reflect the complexity of results presented, and this should be amended.

*Response: We have amended this in the results (page 9; see below) and improved the discussion section (see above) to account for these discrepancies.*

*Text added in results section (page 9):*

*Taken together, these results suggest a complex interplay between H3.3 mutations, ATRX, and PML. Although there is not a strict relationship between onco-histone H3.3 and PML occupancy, it is clear that H3.3 mutations affect the formation of PML-NBs and the localisation of PML. The exact effects appear to vary depending on the specific mutation (K27M/G34R) and the presence of ATRX. Nonetheless, all the tested mutations resulted in increased H3.3 incorporation at promoters which could* *trigger a self-reinforcing feedback loop where disruption of PML leads to increased incorporation of mutant H3.3.*

7) Figure 3 - In the case of tumor-derived cell lines, the authors observe opposite effect when compared to mESCs results when it comes to number of PML-NBs - they increased in mESCs and decreased in the tumor-derived lines. In both cases however the levels of H3.3 increases over the promoter - it would be important to know how do the PML levels change in these regions? Doesn't the fact that changes in H3.3 and PML-NBs are not well correlated with each other argue that these two factors are not strictly related?

*Response: Although PML-NBs are more numerous in H3.3 K27M mutated mouse ES cells, these PML-NBs are much smaller compared to WT cells (****Fig. 2A-D****), indicating that they may not be functional. This would yield an equivalent phenotype to the loss of PML-NBs seen in the tumour-derived cell lines. We are not able to address this directly at present since molecular characterisation of PML-NBs is quite technically challenging. In addition, there are currently no PML antibodies suitable for ChIP in human cells. We have attempted PML ChIP-seq several times with different antibodies but have not yet been successful.*

*We agree with the reviewer that there is not a strict relationship between H3.3 and PML-NBs. In addition to the ATRX/DAXX complex which clearly localises to PML-NBs, H3.3 is also associated with the HIRA chaperone complex that targets H3.3 to active genes, which is not strictly linked to PML. This is a complex pathway which doesn’t interact in a direct linear fashion. We have attempted to emphasise this point more clearly in the revised results and discussion sections pertaining to these figures.*

8) Figure 4 - here the quantification of the phenotype "number of PML bodies" will be necessary - based on the representative images provided it seems like there is a rather strong phenotype of increased number of PML-NBs in H3.1 K27M cell lines as compared to WT H3.3 lines - is this really the case? A great deal of effort was placed in previous panels to document the PML-NBs changes in H3.3 mutants and as is, it is not convincing to rule out any differences in H3.1 mutant based on unquantified images only.

*Response: We have provided the quantification of the phenotype "number of PML bodies" in* ***Fig. 4C****. Unlike H3.3 K27M cell lines, H3.1 K27M cell lines showed higher number pf PML-NBs. We have included these results and discussed this in the text.*

*Text added in results section (page 11):*

*Immunohistochemistry staining for PML in H3.1 K27M mutants showed punctate and numerous PML-NBs similar to H3.3 WT (K27M KO) cells. While both H3.3 K27M mutants (DIPG XIII median = 4; BT245 median = 4, n = 50) had reduced numbers of PML-NBs relative to WT H3.3 (K27M KO) (DIPG XIII median = 14, BT245 median = 11, n = 50, P < 0.0001) (Fig. 4A, C, S4B), the H3.1 K27M mutants had higher numbers of PML-NBs on average (median #1-#4 = 22; 16.5; 14.5; 20; n = 50; H3.3 K27M aggregate median = 22, n = 200, P < 0.0001) compared to the WT H3.3 (K27M KO) lines (Fig. 4B, C, S4B). As there are no genuine “WT” representative of these cells, it is not possible definitively determine if the H3.1 mutations truly increase PML-NBs relative to a true WT population. It is nonetheless clear that H3.3 and H3.1 K27M mutations have distinct effects on PML-NBs. The loss of PML-NBs is an H3.3-specific phenomenon and suggests that PML disruptions can contribute to oncogenesis in H3.3-mutated paediatric gliomas.*

9) Figure 5 - the fact that depletion of PML leads to failure in differentiation is a strong and suggestive result; however, it does not directly link oncohistone tumor-derived cells phenotype to disruption of PML bodies; taking into account that in tumor-derived cells the number and size of PML-NBs indeed decrease, maybe rescuing the non-differentiation phenotype in these cells via PML overexpression would be a better suited experiment, as it could provide direct evidence that oncohistone-driven phenotype are indeed linked to its effect on PML-bodies.

*Response: We thank the reviewer for this suggestion. We have now overexpressed PML in H3.3 WT and H3.3 K27M mutated DIPG XIII glioma cells and assessed these lines for both differentiation capacity by staining for GFAP expression (****Fig. 5C-D****) and arsenic sensitivity (****Fig. S6 G-H****). As anticipated, PML overexpression was indeed able to partially rescue the differentiation phenotype and reduced arsenic sensitivity in the H3.3 K27M mutant cells. This strengthens our hypothesis and provides robust evidence that the H3.3 K27M mutation act through disruptions of PML bodies.*

**Figure 5 C-D**

**Figure S6G-H**

10) Figure 6 - this is an interesting experiment in light of Figure 5 - if the authors are correct, that the therapeutic effect of ATO in the case of oncohistone tumor-derived cells is due to degradation of PML, then the PML KD experiment should give rise to similar results in a cell viability assay - is that the case? That would be an additional and strong evidence that cell fate aberrations in oncohistone tumor-derived cell lines are linked with PML function.

*Response: While functional PML-NBs are required for neural differentiation (****Fig. 5****), the PML response to ATO is somewhat more complex (****Fig. 6****). Previous studies have shown that ATO has a dual effect in PML-RARα mutated APL – first promoting PML assembly and then subsequent degradation through hypersumoylation. Importantly, the assembly of PML-NBs is thought to be important for the effectiveness of ATO, as mutations in the PML binding sites disrupt PML-NB assembly and blunt the ATO response.*

*We find a similar response in the histone-mutated DIPG lines, where ATO first triggers increased PML in insoluble fractions followed by degradation (see western blots below;* ***Fig. S6 A-B****). Given that PML-NBs are insoluble, we altered the experiments to capture these fractions and observed increased PML in S3 and the pellet fractions at 4 hours post-ATO treatment in both K27M and K27M KO controls, which was followed by loss of PML at 24 hours in the K27M DIPG cells. Similar observations were made with PML-NB immunostaining (****Fig. S6 C-D****), where increased PML-NBs were seen following ATO treatment, and the staining intensities were lost after 24hr of ATO treatment. We have added this to the results and discussion sections and clarified the mechanism of action for ATO.*

*Given that ATO response in APL is thought to be dependent on PML-NB assembly, it is unlikely that PML KO has an equivalent effect to ATO.*

**Figure S6A-B**

**Reviewer #2 comments:**

The manuscript by Voon et al. discusses the point mutations in H3.3 disrupt the formation of PML nuclear bodies and prevents differentiation down glial lineages. The tumour cells carrying H3.3 mutations are sensitive to drugs that target PML bodies. The point mutations in IDH1/2 are also associated with disrupting the formation of PML bodies. The manuscript is well written and attractive.

*Response: We thank the reviewer for being positive to our paper.*

Specific comments:

1) The authors demonstrated that H3.3 K27M patient-derived glioma cells are sensitive to ATO through in vitro experiment. Then, how about the results by in vivo assay?

*Response: Although it may have benefit to assess sensitivity of H3.3 K27M patient-derived glioma cells to ATO in vivo (such as in a xenograft mouse model), the result is unlikely going to change the conclusion of our findings. Addressing an effect of PML targeting in patients would of course be extremely informative, however, it is unclear how this could (safely) be performed; this is an entirely different ballpark from what we are reporting here. Importantly, we would like to emphasize that we have now included several additional H3.3 K27M patient-derived cell lines in our revisions (****Fig. S6 C-D****), and all they have shown sensitivity to ATO. Thus, even though we concede that taking the next step to an in vivo context would be the ultimate, we believe our results provide significant new information on the possibility to target PML as a potential therapeutic approach.*

**Figure S6 C-D**

2) As mentioned by the authors that IDH1 mutation could have an influence on PML-NBs, and IDH1 mutated cells were likely to be sensitive to treatment with ATO. So, it would be interesting to compare the response of the cell lines with H3.3 mutation or IDH1 mutation to ATO treatment.

*Response: This point is duly noted. We have provided data on ATO treatment of a glioma cell line carrying an IDH1 mutation, and cells with IDH1 mutation are more sensitive to ATO treatment when compared to their WT isogenic control cells (****Fig. 7F-G****). Although IDH1 mutation has influence on PML-NBs, it is not a main focus of the paper to determine its sensitivity to ATO. We would strongly prefer to not compare the responses of cell lines with H3.3 mutations or IDH1 mutations to ATO treatment as it would distract from our main focus, which is mutations in H3.3.*

3) The figures are unclear especially in figure 6 and figure 7.

*Response: We’re afraid we are not sure what Reviewer #2 is referring to here. Since reviewer 1 has no comment on the “clarity” of our figures, we have opted for not altering anything beyond what was required by the actual revision process.*

***References***

*1	Corpet, A. et al. PML nuclear bodies and chromatin dynamics: catch me if you can! Nucleic Acids Res* ***48****, 11890-11912, doi:10.1093/nar/gkaa828 (2020).*

*2	Lallemand-Breitenbach, V. & de The, H. PML nuclear bodies: from architecture to function. Curr Opin Cell Biol* ***52****, 154-161, doi:10.1016/j.ceb.2018.03.011 (2018).*

*3	Niwa-Kawakita, M. et al. PML is a ROS sensor activating p53 upon oxidative stress. J Exp Med* ***214****, 3197-3206, doi:10.1084/jem.20160301 (2017).*

*4	Ablain, J. et al. Activation of a promyelocytic leukemia-tumor protein 53 axis underlies acute promyelocytic leukemia cure. Nat Med* ***20****, 167-174, doi:10.1038/nm.3441 (2014).*

*5	Koken, M. H. et al. The PML growth-suppressor has an altered expression in human oncogenesis. Oncogene* ***10****, 1315-1324 (1995).*

*6	Terris, B. et al. PML nuclear bodies are general targets for inflammation and cell proliferation. Cancer Res* ***55****, 1590-1597 (1995).*

*7	Hodges, M., Tissot, C., Howe, K., Grimwade, D. & Freemont, P. S. Structure, organization, and dynamics of promyelocytic leukemia protein nuclear bodies. Am J Hum Genet* ***63****, 297-304, doi:10.1086/301991 (1998).*

*8	Lallemand-Breitenbach, V. et al. Arsenic degrades PML or PML-RARalpha through a SUMO-triggered RNF4/ubiquitin-mediated pathway. Nat Cell Biol* ***10****, 547-555, doi:10.1038/ncb1717 (2008).*

*9	Lallemand-Breitenbach, V. et al. Role of promyelocytic leukemia (PML) sumolation in nuclear body formation, 11S proteasome recruitment, and As2O3-induced PML or PML/retinoic acid receptor alpha degradation. J Exp Med* ***193****, 1361-1371, doi:10.1084/jem.193.12.1361 (2001).*

*10	Jeanne, M. et al. PML/RARA oxidation and arsenic binding initiate the antileukemia response of As2O3. Cancer Cell* ***18****, 88-98, doi:10.1016/j.ccr.2010.06.003 (2010).*

*11	Rerolle, D. & de The, H. The PML hub: An emerging actor of leukemia therapies. J Exp Med* ***220****, doi:10.1084/jem.20221213 (2023).*

*12	Weidtkamp-Peters, S. et al. Dynamics of component exchange at PML nuclear bodies. J Cell Sci* ***121****, 2731-2743, doi:10.1242/jcs.031922 (2008).*
